# Beyond standardized mortality ratios; some uses of smoothed age-specific mortality rates on small areas studies

**DOI:** 10.1186/s12942-020-00251-z

**Published:** 2020-12-04

**Authors:** Jordi Perez-Panades, Paloma Botella-Rocamora, Miguel Angel Martinez-Beneito

**Affiliations:** 1 Direcció General de Salut Pública i Addiccions, Conselleria de Sanitat Universal i Salut Pública, Avda/Cataluña, 21, 46020 Valencia, Spain; 2 Departament d’Estadística i Investigació Operativa, Universitat de València, C/Dr. Moliner, 50, 46100 Burjassot, Valencia Spain

**Keywords:** Disease mapping, Age-specific smoothing, Life expectancy, Age-standardized rates

## Abstract

**Background:**

Most epidemiological risk indicators strongly depend on the age composition of populations, which makes the direct comparison of raw (unstandardized) indicators misleading because of the different age structures of the spatial units of study. Age-standardized rates (ASR) are a common solution for overcoming this confusing effect. The main drawback of ASRs is that they depend on age-specific rates which, when working with small areas, are often based on very few, or no, observed cases for most age groups. A similar effect occurs with life expectancy at birth and many more epidemiological indicators, which makes standardized mortality ratios (SMR) the omnipresent risk indicator for small areas epidemiologic studies.

**Methods:**

To deal with this issue, a multivariate smoothing model, the **M**-model, is proposed in order to fit the age-specific probabilities of death (PoDs) for each spatial unit, which assumes dependence between closer age groups and spatial units. This age–space dependence structure enables information to be transferred between neighboring consecutive age groups and neighboring areas, at the same time, providing more reliable age-specific PoDs estimates.

**Results:**

Three case studies are presented to illustrate the wide range of applications that smoothed age specific PoDs have in practice . The first case study shows the application of the model to a geographical study of lung cancer mortality in women. This study illustrates the convenience of considering age–space interactions in geographical studies and to explore the different spatial risk patterns shown by the different age groups. Second, the model is also applied to the study of ischaemic heart disease mortality in women in two cities at the census tract level. Smoothed age-standardized rates are derived and compared for the census tracts of both cities, illustrating some advantages of this mortality indicator over traditional SMRs. In the latest case study, the model is applied to estimate smoothed life expectancy (LE), which is the most widely used synthetic indicator for characterizing overall mortality differences when (not so small) spatial units are considered.

**Conclusion:**

Our age–space model is an appropriate and flexible proposal that provides more reliable estimates of the probabilities of death, which allow the calculation of enhanced epidemiological indicators (smoothed ASR, smoothed LE), thus providing alternatives to traditional SMR-based studies of small areas.

## Background

Spatial epidemiology deals with the description and analysis of geographically indexed health data with respect to demographic, environmental, behavioral, socioeconomic, genetic and infectious risk factors [1]. Most of these risk factors, especially those related to mortality, strongly depend on age [2]; therefore, the comparison of raw (unstandardized) rates may be misleading due to differences in the population structure of the units of study, which may be confused with real differences in risk. Age-adjusted (standardized) rates were proposed in the mid-nineteenth century [3] as an answer to this problem, although they are not a completely satisfactory solution.

Standardization seeks to remove the effect of having different age distributions for the populations being compared. It produces a single summary index per population, easier to compare than a full set of age-specific rates. The most popular standardization method is the so-called indirect method, which would yield standardized mortality ratios (SMRs) if mortality was the event of interest. SMRs are calculated as the ratio of the number of deaths observed over a specific time interval in a population group, in our case and from now on “spatial units”, to the expected deaths in that population assuming that it had the same age-specific death rates as a reference population. This indicator is commonly used to compare the mortality for different geographical areas to that of the reference population, and its main advantage is that SMRs do not depend on the age-specific rates of each spatial unit, which may be completely unreliable when working with small areas. This property has led to SMRs being, by far, the most commonly used epidemiological indicator for small areas spatial studies. Nevertheless, SMRs do exhibit some particular problems. SMRs of different spatial units are not directly comparable, that is, even if the age-specific rates of two populations were all proportional, this would not mean that the same proportionality holds between their SMRs [4]. In particular, areas with identical age-specific rates do not necessarily yield the same SMRs. Therefore, strictly speaking, SMRs are only valid for comparing the units of study against the standard population of the study, but not against one another.

Alternatively, a second age-standardization method for rates, the direct method, produces age-standardized rates (ASRs), a weighted average of the age-specific rates of each unit of study also using a reference population to determine that common set of weights. When mortality is the outcome of study, ASRs have a direct interpretation as the hypothetical death rate that would have occurred in the corresponding unit of study if its age composition were the same as that of the reference population [5]. Therefore, the standardization process of ASRs removes the effect that the age composition of the different population groups could have on raw rates. One advantage of direct over indirect standardization is that spatial units with proportional age-specific rates necessarily yield ASRs keeping that same proportion [4], and so they are also comparable to each other. Nevertheless, ASRs are not as popular as SMRs for small areas geographical analyses since ASRs are only reliable when trustworthy age-specific rates are available for each spatial unit. This usually requires having a considerable amount of observed health events, from now on “deaths”, for every combination of age group and spatial unit, which small areas do not usually have. As a consequence, when ASRs are pursued on small areas, statistical modeling becomes necessary.

There have been various approaches to the smooth estimation of ratios, such as mortality rates. In this regard, Kafadar [6] proposed a linear smoother for geographically-defined data that are in form of ratios. Although it shows that the smoothed rate has less variability than the age-adjusted unsmoothed rate, it uses parametric weights and does not consider the dependence between age groups. Ezzati et al. [7] used information on the number of deaths and county-level sociodemographic characteristics to estimate mortality rates and probabilities of death, but merged the counties, pooled the data on deaths and population over 5 years and also did not consider the dependence between age groups.

Many more epidemiologic measures show similar problems produced by the age composition of the units of study. For example, life expectancy at birth (LE) is an alternative mortality summary which shows similar problems. This indicator has the advantage of being very intuitive and easier to understand for the majority of people, therefore its use is increasingly demanded from health authorities. Its calculation also depends on the age distribution of mortality for each unit of study. Thus, although directly comparable between spatial units, it also poses the small area estimation problems arising from the age-specific risk estimates for each spatial unit [8]. Several Bayesian hierarchical approaches have been previously proposed to estimate smoothed LEs in small areas studies. Congdon [9] considers the smoothing of LEs but his model does not contain a specific age–space interaction term, so age–space risk variations are just a simple sum of separate age and space components. In other works [10–14] model area and age specific mortality rates in order to subsequently calculate LEs; nevertheless, their approaches do not include a fully structured (dependent) age–space interaction term jointly considering the variation of these two sources of dependence. These works do not use the dependence of contiguous age groups and locations for the age–space interaction term. As a consequence, these models fit the underlying overdispersion but could possibly fail to appropriately describe the space–age group structured interaction of the data. Consequently, the technical difficulties of structuring the dependence structure in age–space terms for LEs have not yet been properly addressed in the literature.

In this paper, an autoregressive proposal, based on the original model developed by Botella-Rocamora et al. [15], is made in order to model the age-specific probabilities of death (PoDs) for each spatial unit, which assumes stronger dependence between closer age groups and spatial units. This age–space dependence structure enables information to be transferred between neighboring (consecutive) age groups and neighboring areas, at the same time, providing more reliable age-specific PoDs estimates. Moreover, the model proposed can be easily generalized to the analysis of age–space–time datasets producing spatio-temporal age-specific rates, for example, or include any additional factor, such as sex, which could be of interest to be studied. The smoothed age-specific PoDs from this model could be later used to derive enhanced indicators, such as smoothed ASRs or LEs, of high epidemiological value.

This article is organized as follows. "Methods" section shows our modeling proposal for estimating age-specific PoDs in small areas geographical studies and its application to the construction of additional smoothed epidemiological indicators such as ASRs and LEs. The following section shows three case studies illustrating some real uses that smoothed age-specific PoDs could have, enhancing therefore traditional SMRs-based small areas studies. The first case study shows the application of the model introduced in "Methods" section to a real setting, a geographical study of lung cancer mortality in women. Its goal is to illustrate the convenience of considering age–space interaction in geographical studies. In the second case, the model is applied to study mortality from ischaemic heart disease for women in two cities at the census tract level. Smoothed ASRs are derived and compared for the census tracts of both cities, illustrating some advantages of this mortality indicator. In the last case study, the model is applied to estimate smoothed LEs, which is the most widely used synthetic indicator for characterizing overall mortality differences throughout areas. Finally, "Discussion" section summarizes the main contributions of our work.

## Methods

### Spatial smoothing of age-specific PoDs

Martinez-Beneito [16] proposed a unifying framework for multivariate disease mapping, i.e., for jointly mapping several diseases taking into account the spatial correlation that they might show. A second reformulation of this proposal, the **M**-model, was proposed in Botella-Rocamora et al. [15] which enables the joint smoothing of tens of diseases in a single study. This approach, with important computational benefits, allows researchers to determine and take advantage of the correlations among diseases, revealing the existence of common risk factors or unknown associations among some diseases. In this model, dependence between spatial units is introduced in a structured way, while the relationship between diseases is introduced in an unstructured way, no prior dependence structure is assumed between diseases. However, this model allows considering also structured relationships between diseases, or geographical patterns in general, (such as an autoregressive structure) if this was considered convenient. Thus, the relationship between geographical patterns of different age groups should be possibly considered as a structured factor, reproducing its ordinal character. This modeling proposal could also be used in our case to model several risk patterns, one per age group, instead of several diseases, although assuming stronger correlations for contiguous age groups. This could be achieved, for example, by assuming spatially dependent first-order autoregressive dependency structures for age within each spatial unit.

Our model proposes to model PoDs instead of age-specific rates. Age-specific rates are the relation between observed deaths and the person-years at risk available. Those person-years are not so easily available for small areas studies, which is why we propose to model PoDs instead of rates. Nevertheless, if mortality rates were really our goal, the person-years in the study could be reasonably approximated by the population at risk for each area multiplied by the years in the period of study, as we will see later. Additionally, LEs depend directly on PoDs instead of rates so the modeling of PoDs will be also suitable for deriving these indicators.

The autoregressive adaptation of the **M**-model for PoDs was made as follows. We changed the typical Poisson likelihood made for most disease mapping studies by a binomial distribution since the population of each (small) spatial unit is further divided into several age groups, which makes the denominator of many age specific PoDs quite low. As a consequence, the binomial assumption seems substantially more appropriate in this context as, in contrast to the Poisson case, it implicitly assumes that the number of deaths for each spatial unit and age group may not be higher than the corresponding population. Thus, let $Y_{sa}$, $N_{sa}$ and $P^*_{sa}$ be the number of observed deaths, population and (unknown) PoDs, respectively, for the *s*-th spatial unit and *a*-th age group, $s=1,\ldots,S$; $a=1,\ldots,A$. We use the asterisk in $P^*_{sa}$ in order to distinguish the smoothed age-specific PoDs that we are modeling from the raw age-specific PoDs, $P_{sa}=Y_{sa}/N_{sa}$. We assume that $Y_{sa}$ follows the binomial distribution:

$$Y_{sa} \sim \text {Binomial}(N_{sa}, P^*_{sa}), \quad s=1,\ldots,S, a=1,\ldots,A,$$

and model the *logit* of the PoDs as:

1 $$\begin{aligned} logit(P^*_{sa}) = \mu _{a} + \theta _{sa} \end{aligned}$$

where $\mu _{a}$ represents the intercept of the *a*-th age group and $\theta _{sa}$ models the PoDs variability between age groups and spatial units. Note that $P^*_{sa}$ in this model, and in general the PoDs that we are referring to, are the conditional probabilities of death at the age interval *a* given that you have survived the previous age intervals.

By modeling $\theta _{sa}$ we will induce dependence between age groups and spatial units at the same time. The ideas of the **M**-model will be used for this end. Specifically, Botella-Rocamora et al. [15] show that it is possible to induce both spatial and multivariate dependence on $\varvec{\Theta }=(\theta _{sa})$ as the product of matrices:

2 $$\begin{aligned} \varvec{\Theta } = \varvec{\Phi } \mathbf{M } \end{aligned}$$

where $\varvec{\Phi }$ is an $S\times A$ matrix whose *a*-th column $\varvec{\Phi }_{\cdot a}$ follows a spatially correlated distribution. In the $\varvec{\Phi }$ matrix, the columns are independent of each other, and therefore they do not consider dependence between groups. The second term in this expression **M**, is an $A\times A$ matrix which induces in $\varvec{\Theta }$ the dependence between age groups that we pursue.

The j-th column of $\varvec{\Theta }$, say $\varvec{\Theta }_{\cdot j}$, contains the spatially referenced logit probabilities for the j-th age group. One can, therefore, interpret that Eq. (2) defines the spatial patterns in $\varvec{\Theta }$ as a linear combination of underlying latent variables whose coefficients correspond to the *j*-th column of **M**. Thus,

3 $$\begin{aligned} \varvec{\Theta }_{\cdot j}= \varvec{\Phi }_{\cdot 1} m_{1j} + \ldots + \varvec{\Phi }_{\cdot A} m_{Aj} \end{aligned}$$

where $m_{ij}$ is the (*i*, *j*)-th entry in **M**. Matrix **M** combines, through linear combinations, the spatial patterns of $\varvec{\Phi }$ yielding therefore dependent spatial patterns.

In particular, we model the set of columns as independent Proper Conditional Auto-Regressive (PCAR) distributions, that is:

$$\begin{aligned} \varvec{\Phi }_{\cdot a} \sim N(0, \sigma ^2 ({\mathbf {D}}- \gamma _a {\mathbf {W}})^{-1}), \end{aligned}$$

where ${\mathbf {W}}$ and ${\mathbf {D}}$ are the adjacency and diagonal matrices typically summarizing the geographical structure in PCAR distributions (see [17] for example).

According to Botella-Rocamora et al. [15], for the separable case with $\gamma _1=\cdots=\gamma _A$, the variance covariance matrix between age groups $\varvec{\Sigma }$ is equal to $\mathbf{M }^T \mathbf{M }$. Therefore, if we want the correlation between age groups to decrease as a function of their distance, **M** should be modelled in order to reproduce that effect. We will assume for simplicity a first-order autoregressive dependence structure between age groups. In that case, the covariance matrix between age groups would take the form:

$$\begin{aligned} \Sigma _{ij} = \rho ^{|i-j|}, \end{aligned}$$

where $\rho$ is an autoregressive dependence parameter controlling the strength of the dependence between age groups. Note that the variance parameter of $\varvec{\Sigma }$ has been removed from this latest expression since the overall variance of $\varvec{\Theta } = \varvec{\Phi } \mathbf{M }$ is already controlled by the term $\varvec{\Phi }$ by means of the variance parameter $\sigma ^2$ of its columns. An **M** matrix inducing the mentioned autoregressive dependence between age groups could be, for instance, the upper-triangular Cholesky matrix of $\varvec{\Sigma }$, which has this simple expression:

$$\begin{aligned} \mathbf{M }= \left( \begin{array}{ccccc} 1 &{} \rho &{} \rho ^2 &{} \cdots &{} \rho ^{A-1}\\ 0 &{} (1-\rho ^2)^{1/2} &{} \rho (1-\rho ^2)^{1/2}&{} \cdots &{} \rho ^{A-2} (1-\rho ^2)^{1/2}\\ 0 &{} 0 &{} (1-\rho ^2)^{1/2} &{} \cdots &{} \rho ^{A-3} (1-\rho ^2)^{1/2}\\ \vdots &{} \vdots &{} \vdots &{} \ddots &{} \vdots \\ 0 &{} 0 &{} 0 &{} \cdots &{} (1-\rho ^2)^{1/2} \end{array} \right) . \end{aligned}$$

To complete the model specification, an improper flat uniform prior is proposed for $\mu _a$ and a uniform prior distribution on the interval $[-1,1]$ for $\rho$. A vague uniform prior on [0, *C*] for a high enough (non-informative) value of *C* is also proposed for the standard deviation $\sigma$ (see Gelman [18] or pages 164–171 of Martinez-Beneito and Botella-Rocamora [17] for more details). Finally, a uniform prior distribution on $]\lambda _{(1)}^{-1},\lambda _{(I)}^{-1}[$, where $\lambda _{(1)}$ and $\lambda _{(I)}$ are the lowest and highest eigenvalues of ${\mathbf {D}}^{-1/2}{\mathbf {W}}{\mathbf {D}}^{-1/2}$ respectively, is proposed for $\gamma _1,\ldots,\gamma _A$, as suggested, for example, by Sun et al. [19] or Martinez-Beneito and Botella-Rocamora [17].

Note that the proposed model is equivalent to the spatio-temporal autoregressive model of Martinez-Beneito et al. [20]. This proposal would model the matrix $\varvec{\Theta }$ as a first order autoregressive process as:

$$\begin{aligned} \varvec{\Theta }_{sa} \sim N(\rho \varvec{\Theta }_{s(a-1)},\sigma ^2_{\varvec{\Theta }}) .\end{aligned}$$

In this case, we would be using the autoregressive component for modeling dependence between age groups instead of temporal dependence. Nevertheless, we have posed our proposal as a **M**-model for several reasons. First, the **M**-model can be easily generalized to non-separable dependence structures; in fact, the model just proposed is already inseparable because of the different spatial correlation parameters ($\gamma _1,\ldots,\gamma _A$) considered for the different spatial terms in the model. In a similar manner, different variance parameters ($\sigma _1^2,\ldots,\sigma _A^2$) could be considered for these patterns, yielding therefore more flexible, heteroscedastic, covariance structures [21]. Second, **M**-models are computationally convenient for modeling multivariate spatial patterns, which makes them an appropriate choice for fitting the data in regular Bayesian inference packages such as WinBUGS, OpenBUGS, Nimble… Finally, and in our opinion most importantly, **M**-models have been generalized to multidimensional models [22] where several factors, besides the spatial component, are considered. This would make it possible to consider additional spatial patterns that could be correlated with the age-specific PoDs that we are modeling in order to enhance the estimates in the model. For example, mortality data from other sexes or causes of death could be additionally considered in order to yield improved age-specific PoDs in our data set. Alternatively, multidimensional modeling could be used to disaggregate the data, for example by subperiods, race groups… while maintaining a reasonable risk estimate quality by considering dependence between these new groups. In summary, in our opinion, all this makes the **M**-models an appropiate and flexible proposal for modeling the age-specific PoDs, as proposed above.

We should mention that some other previous proposals in the literature have dealt with the joint analysis of several dependence sources, such as age–space–time models. For example, Goicoa et al. [24] proposed an age–space–time separable model where age and time are modelled as first order random walks and spatial dependence is modelled by means of a Leroux et al. [23] dependence structure. Our proposal yields, in principle, more flexible dependence structures since different spatial correlations, for example, for each underlying spatial pattern are considered. Moreover, the autoregressive dependence structure for age groups allows the strength of this dependence source to be adapted. These modelling features could be implemented in INLA (the software package used in [24]), which in principle could take advantage of the sparse structure of the spatial and temporal precision matrices to speed up computations [25]. Nevertheless, this would not be so advisable, since those modelling features would increase the number of parameters to be integrated out by INLA, what would make its fit substantially slower. Similarly, Goicoa et al. [26] propose alternative age–space–time models, but using P-splines for modeling dependence for age and time. Once again, considering spatial random effects of different dependence parameters would seem problematic for this work as well. Thus, in this regard we find our proposal somewhat more suitable. Moreover, although some other Markov chain Monte Carlo (MCMC) based approaches have been proposed for the modeling of age–space–time interactions [27] we find our proposal particularly convenient as it does not require a specific coding of the MCMC algorithm and, in contrast, it can be fitted with regular Bayesian software packages.

### Epidemiological applications of the smoothed age-specific PoDs

The proposal above produces age-specific PoDs, $P^*_{sa}$, smoothed over space and age groups. It is important to note that any statistical indicator built on ${\mathbf {P}}^*=(P^*_{sa})$ will inherit the smooth character of ${\mathbf {P}}^*$, thereby producing smooth, and in principle more reliable, estimates of other more complex elaborate epidemiological indicators.

For example, ASRs are weighted averages of the age-specific death rates. The ASRs weights would be given by the age-composition of a reference population, so all the ASRs would represent the observed risks for a common (ideal) population; in this way the age composition of the units of study would in principle no longer be a confounding factor. Traditionally, the age-specific mortality rate for age group *a* and spatial unit *s* is given by:

4 $$\begin{aligned} R_{sa} = \frac{Y_{sa}}{PersonYears_{sa}}\approx \frac{Y_{sa}}{T \cdot N_{sa}}=\frac{P_{sa}}{T} . \end{aligned}$$

where *T* is the number of years in the period of study. If the period of study was just one year then $T=1$ and then $R_{sa}=P_{sa}$. Let $pob_a$ be the population in age group *a* in the reference population and let the standard weights be given by:

$$\begin{aligned} W_a=10^5 \cdot \frac{pob_a}{\sum _a pob_a}. \end{aligned}$$

Then the ASR for the *s*-th spatial unit is usually defined as:

5 $$\begin{aligned} ASR_{s}=\sum _a W_a \cdot R_{sa}. \end{aligned}$$

The $10^5$ in $W_a$ allows us to undertand the ASRs as the number of deaths that we would expect per 100,000 people from the reference population. Obviously, the unreliability of $P_{sa}$ when the spatial units are small will be transferred to the ASRs, which will make them unreliable indicators. However, we could alternatively define smoothed Age-Standardized Rates (sASRs) by simply replacing $R_{sa}$ in the previous expression by $R_{sa}^*=P_{sa}^*/T$, that is:

6 $$\begin{aligned} sASR_{sa}=\sum _a W_a \cdot R^*_{sa} \end{aligned}$$

which will inherit the smooth character of $P^*_{sa}$ and will therefore solve the small area estimation problems that traditional ASRs show.

Following this same idea, many more epidemiological indicators relying on age-specific PoDs or mortality rates could be smoothed. For example, LEs are usually calculated from life tables as a direct function of the age-specific PoDs, the width of each age interval and the fraction of the last age interval survived for those people dying at each age interval [28]. Additionally, particular care is required for the last age group since this is a right-opened interval, with no upper limit, so it deserves special attention. Nevertheless, if $D_{sa}$ is the marginal probability of death (which is a combination of the conditional PoDs) for group age *a* and spatial unit *s*, and $age_a$ is the mean age of death for the people dying at age group *a*, LEs could be alternatively defined as the expected value of the observed ages of death in the following way:

7 $$\begin{aligned} LE_s=\sum _{a=1}^{A} age_a D_{sa}. \end{aligned}$$

In a similar manner to that made with life tables, $age_a$ could be calculated as the sum of the initial age of each interval and of the proportion of interval survived by those dying during that age interval. Usually, that proportion survived is assumed to be one half of each interval, except for the youngest age group where it is known that perinatal deaths make this proportion shorter [29]. As a consequence, $age_a$ is usually taken as the central value for each age interval, which is equivalent to assuming the deaths to be uniformly distributed over those intervals. Regarding $age_A$, it is usual to assume a constant death rate, or equivalently an exponential distribution for the ages of death in the latest age group [30]. According to the available data, that death rate could be estimated as the age-specific death rate for this group. Therefore, according to the exponential assumption for the ages of death for this interval, the average number of years lived for any person reaching the oldest age group would be the inverse of its death rate, that is: $T/P_{sA}$. Therefore, $age_A$ would be the sum of this quantity and the initial age for this age group.

A detailed description of the life expectancy calculation from a life table can be found, for example, in the Public Health England template [31] produced for this purpose based on the methodology described in [29].

Once the $age_a$ values have been calculated, Expression (7) can be formulated as a function of the conditional probabilities of death:

8 $$\begin{aligned} LE_s=\sum _{a=1}^{A-1} age_a \left( \prod _{i<a} (1-P_{si}) P_{sa} \right) + age_A \prod _{i<A} (1-P_{si}). \end{aligned}$$

The reliance of Expression (8) on the observed raw age-specific (conditional) PoDs, $P_{sa}$, makes it clear why LEs become unreliable in small areas studies. Nevertheless, by changing the raw age-specific PoDs for the smoothed age-specific PoDs, $P^*_{sa}$, resulting from the above model, we could obtain reliable life expectancies, smoothed Life Expectancy (sLE), suitable for small areas studies.

Similar procedures could be proposed for any other epidemiological indicator relying on age-specific PoDs, such as disability-free life expectancy, potential years of life lost… to cite just two. These indicators would be alternative tools to the traditional use of SMRs in small areas studies that would highlight new aspects of the data that regular SMRs are not able to reveal.

Finally, we find it interesting to note that, since the proposed model is posed under a Bayesian approach, MCMC is used for making inference about that model. As a consequence, credible intervals and other additional variability measures can be easily computed within the MCMC itself for any of the proposed indicators. This makes their comparison particularly easy.

## Case studies

In this section, we are going to make use of some real case studies to illustrate the use of the proposed methodology and the advantages of the new smoothed epidemiological indicators over the traditional use of smoothed SMRs, from now on “sSMRs”.

All the models used for the following case studies have been run in WinBUGS [32]. The pbugs [33] R library was used for speeding up computations by parallelizing the sampling of the different chains of the MCMC. Rmarkdown documents with the code used to reproduce all three analyses can be found at Additional file 1. For all three analyses, three chains were run with 30,000 iterations, the first 5000 of which were discarded as a burn-in period. A thinned sample was obtained by saving one out of every 75 iterations. Regarding convergence checking, the Brooks–Gelman–Rubin statistic [34] has been checked to be lower than 1.1 and the effective sample size [35] to be above 100 for all the parameters saved during the MCMC. All these criteria were successfully met for all three case studies.

All three models that were finally run for all three case studies correspond to separable age–space dependence structures, with $\gamma _1=\cdots=\gamma _A$. The reason for this is that for none of the three data sets have we found evidence of inseparability (the confidence intervals for $\gamma _1,\ldots,\gamma _A$ for all three studies substantially overlapped). As a consequence, we have decided to show the results of the simpler separable case. However, we should mention that we have found evidence of inseparability for some other case studies that have not been finally included in the paper for reasons of space.

### Case study 1: interaction between age groups and space

In this first case study, the proposed model has been applied to the study of lung cancer mortality in women in Comunitat Valenciana (Spain) for the 2008–2017 period. Municipalities were the spatial units of study, with a total of 542 municipalities composing the whole Comunitat Valenciana (CV). The average number of women in CV during the period of study is around 2.5 millions. The mean number of women per municipality is 4695 while the corresponding median is 744. Nowadays, lung cancer is the second most common cancer mortality cause in women in CV after breast cancer, with a total of 4232 deaths for the whole period of study. Mortality data were provided by the mortality registry of CV. Population data was obtained from the Spanish National Statistics Institute. Both sources of information are considered to have good data quality at this level of disaggregation. It has not been necessary to process missing data or other data quality issues.

Since lung cancer mainly affects elderly people, the younger age groups have been merged, resulting in the following 11 age intervals: [0, 40), [40, 44), [45, 50), …, [80, 85) and [85,…). As a consequence, we have 5962 ($=11\cdot 542)$ age-specific PoDs to estimate, more than observed deaths. Therefore, even though lung cancer shows very high mortality values for our data set, we still have 4646 cells out of the 5962 municipality-age group combinations (78%) with zero deaths. This makes evident the need for some kind of modeling in order to derive reliable results.

**Fig. 1:**
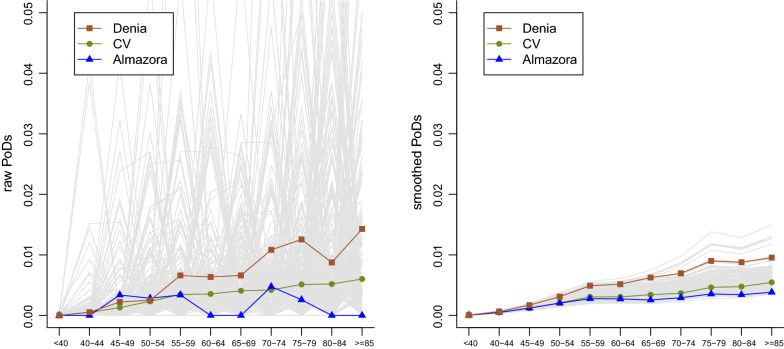
Comparison between raw and smoothed probabilities of death (PoDs) for each area from lung cancer in women in 2008–2017. Denia and Almazora are two illustrative municipalities. Comunitat Valenciana, CV, is the whole study region

The left hand side of Fig. 1 shows the raw PoDs for each area and age group directly obtained from 4. Note that age-specific rates would be just the PoDs divided by 10, the number of years of the period of study. The right hand side of Fig. 1 shows the corresponding smoothed PoDs provided by the model. In both cases, the age-specific PoDs for the whole CV, Almazora and Denia (two illustrative municipalities) have been highlighted. The raw PoDs show evidence of small area estimation problems. There are no observed cases for most age groups and municipalities and when some death is observed this makes the probability of death appear extremely high, mainly for the youngest age groups, which produces highly volatile estimates and sawtooth-shaped curves for most municipalities. These extreme raw age-specific PoDs would evidently distort subsequent epidemiological indicators based on them. On the other hand, the estimated smoothed PoDs, using information from neighboring age groups and areas, yield a much more stable and credible performance where most of the uncertainty in the raw PoDs has been filtered out. Nevertheless, some variability between municipalities is retained, for example, several municipalities stand out in this second plot for having particularly high risks for the oldest groups. These municipalities were completely unnoticed in the left hand side plot where the underlying noise does not allow conclusions of any kind to be drawn.

In order to confirm the relevance of the age–space interaction for this data set, we have run a second alternative model without interaction. In other words, this second model assumes simply $logit(P^*_{sa})=\mu _a+\theta _s$ instead of having a $\theta _{sa}$ term that varies for each spatial unit and age group. We have compared the Deviance Information Criterion (DIC) [36] for both models, resulting in a DIC of 6546.5 for the model without interaction and 6508.0 for the original model with interaction. Therefore, age–space interaction seems required in order to appropriately describe the variability underlying this data set. Although some additional model selection criteria could be used for this comparison, as suggested for example by Duncan and Mergensen [37], the magnitude of the DIC difference already points out important fit differences between both models.

**Fig. 2:**
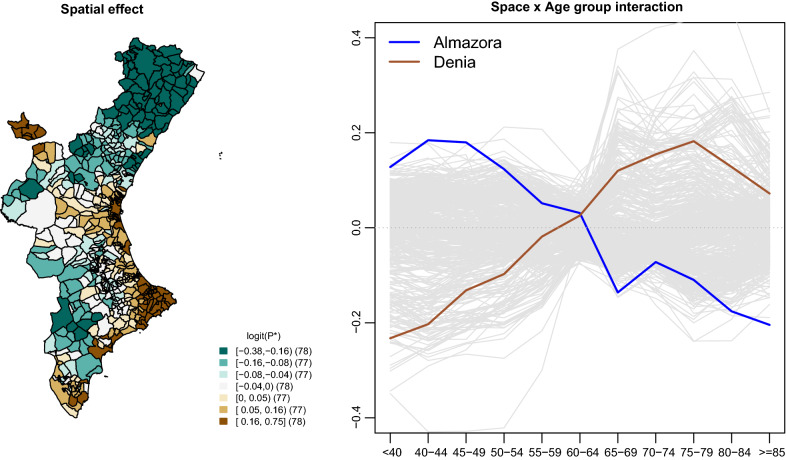
Spatial and interaction terms for the smoothed age-specific PoDs for each area. Denia and Almazora are two illustrative municipalities

We have decomposed the variability on $logit(P^*_{sa})$ into three separate terms in order to visualize the sources of variability of this data set. Specifically, we have decomposed that matrix into two overall age and spatial effects and an interaction term explaining the variability that cannot be explained by these main terms (see Adín et al. [38] or page 285 of Martinez-Beneito and Botella-Rocamora [17] for more details on this decomposition in a spatio temporal setting). The age effect (result shown in Additional file 1), which is defined as the collection of column means of the matrix $\varvec{\Theta }$, unsurprisingly shows an upwards trend of the risks as a function of age, with a steeper trend until the [55, 59] group and a milder constant increase for the oldest age groups. Figure 2 shows both the spatial and interaction terms for each municipality. The spatial term, in the left hand side plot, which is defined as the collection of row means of the matrix $\varvec{\Theta }$, shows a clear spatial pattern, with high risks mainly in the southern and south-eastern part of CV (this was already described at Zurriaga et al. [39]). The cuts for the groups in this plot correspond to septiles of the spatial term, which is in the logits scale. The right hand side plot shows the interaction term for each municipality (one line per municipality). The comparison of the scales of both plots shows that substantial variability still holds for the interaction term, with a range of values comparable to that of the spatial term. As a consequence, particular risk excesses for some age groups and municipalities are as important as the overall risk excesses (for all age groups as a whole) for those same municipalities. In particular, Almazora and Denia show opposing performances in age specific terms. Specifically, Denia exhibits particularly low risks for the youngest age groups while the oldest groups show particularly high risks. On the contrary, Almazora shows the opposite, exhibiting better performance for the older age groups.

**Fig. 3:**
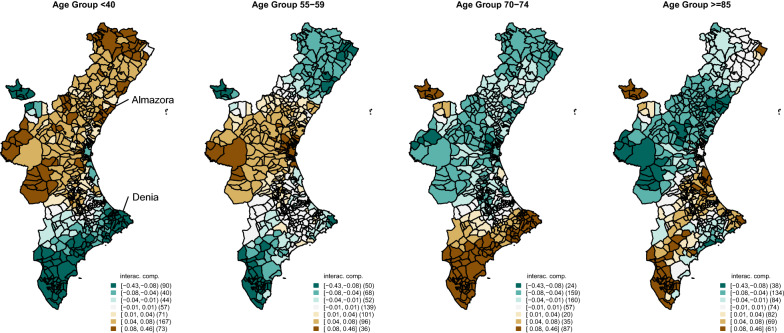
Age–space interaction for several of the age groups considered

Figure 3 shows choropleth maps for the interaction term for several of the age groups considered. Almazora and Denia have been highlighted in the first of these plots. We can see how, in general, the younger groups have a particularly bad performance in the northern and western parts of the CV. In contrast, these same areas have a particularly good performance in the oldest age groups. Note the similarity of the areas shown in this graph with those also outstanding for the spatial term. In particular, the correlation of the spatial component and the interaction term for the group younger than 40 is − 0.65; 0.42 for the age group [70, 74] and 0.32 for the age group older than 85. As a consequence, the combination of the spatial and interaction terms for the younger groups (result not shown) produces a much flatter spatial pattern than that of the spatial term, while that spatial pattern is reinforced with the interaction term for the oldest age groups, which is similar to the spatial pattern in Fig. 2. Therefore, the geographical pattern arising in the spatial term of this analysis is mainly the effect of the risk patterns corresponding to the oldest age groups since that spatial distribution for the younger groups is substantially flatter. This kind of result cannot be obtained from the typical sSMRs analyses usually made by default when studies are carried out on sets of small areas.

### Case study 2: smoothed ASRs

In this case we have applied the model in "Methods" section to the study of ischaemic heart disease (IHD) mortality for women aged over 45 years, during the period 1996–2015. Mortality data were provided by the MEDEA3 Project (“Socio-economic and environmental inequalities in the geographical distribution of mortality in large cities in Spain (1996–2015)”, PI16/01004). Population data was obtained from the Spanish National Statistics Institute. Nine age groups: [45, 50), [50, 55), …, [80, 85) and [85,…) were considered for this study. Two separate studies, with independent models, were carried out to analyse IHD mortality in the two largest CV cities: Valencia and Alicante.

In this case, the spatial unit of study were census tracts, with a total of 531 and 178 units for Valencia and Alicante, respectively.

**Fig. 4:**
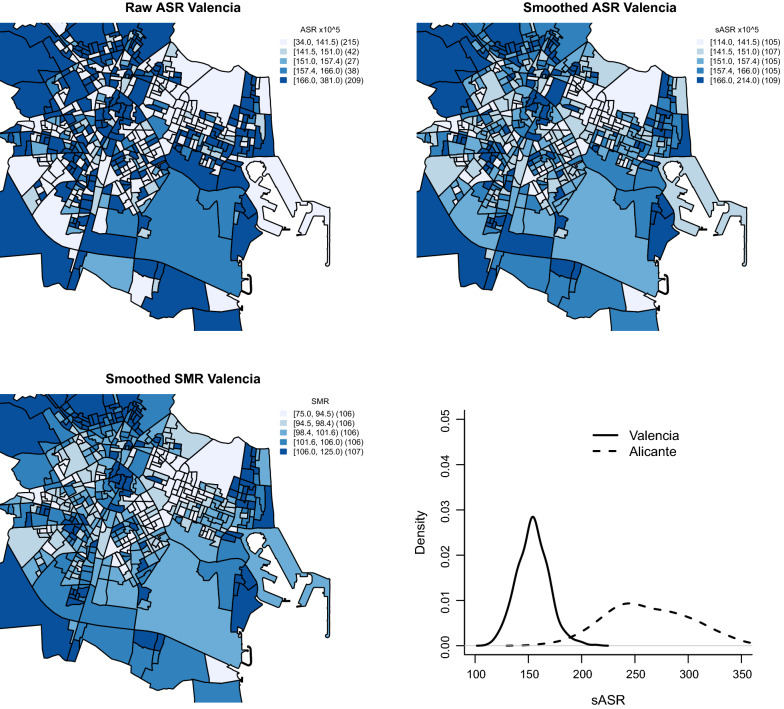
Smoothed age-standardized mortality rate from ischaemic heart disease in Valencia and Alicante. Women over 45 years, period 1996–2015

In this case study, we explore the use of sASRs as an alternative to sSMRs for small areas geographical analyses. Figure 4 shows several choropleth maps summarizing the main results for this analysis. Specifically, the top-left plot in that figure shows the raw ASRs for the census tracts of Valencia. This plot shows substantial noise, which hardly allows any meaningful conclusion to be drawn. Note that the census tract with the highest raw ASR is estimated to have a risk more than 10 times (!) higher than that with the lowest raw ASR. In the top-right plot we show the sASRs calculated as described in the previous section. In contrast to the raw ASRs, the sASRs show a clearer pattern where some neighborhoods show higher risks. In this way, the benefit of using smoothed epidemiological indicators becomes clear. For comparability, quintiles of the Valencia sASRs have been used as cuts for both ASR maps in the top row of Fig. 4. The bottom-left plot of Fig. 4 shows, as a reference, the sSMRs drawn from a typical small area study fitting the [40] model. Quintiles of the sSMRs have been used for cutting the categories for this choropleth map. Note that, despite some small differences, both sSMR and sASR choropleth maps resemble each other, since both are summarizing the mortality risks of the same data set in different manners. In fact, the correlation between both quantities for the Valencia census tracts is 0.95.

Despite the resemblance of the sSMR and sASR maps, the ASRs shows some advantages over the traditional sSMRs that would be appropiate to value. In contrast to sSMRs, sASRs are comparable for the different census tracts of a city, so they could be used, for example, for comparing the risks of two particular census tracts. Thus, the probability that the Valencian census tract with the highest sASR (posterior mean) has a sASR higher than that with the lowest sASR (posterior mean) is virtually 1 for our data set, highlighting an important spatial variability. This statistic should be more powerful than the typical $P(sSMR>100)$ used in many traditional disease mapping analyses since the mentioned sASR statistic takes into account the two extremes of the sASRs distribution instead of comparing the risk of each census tract with that of the reference population.

Additionally, the sASRs calculated could be further compared to other additional ASRs corresponding to other areas of study. Thus, our sASR estimates could be compared for example to the ASR for the overall Valencian Region, Spain or even ASRs corresponding to other countries. In this manner, the use and scope of the generated results would be much wider than for SMRs. As an example, the bottom-right plot of Fig. 4 shows the distributions of the sASRs (their posterior means) for the Valencia and Alicante census tracts. As shown in that figure, most of the Valencia census tracts have sASR values smaller than any of the Alicante census tracts. As a matter of percentiles, the 95% percentile of the sASR distribution in Valencia (178.9 deaths per $10^5$ women) is lower than the 5% percentile for Alicante (203.5). In other words, the census tracts with the highest IHD sASRs in Valencia have comparable mortality risks to those census tracts with the lowest mortality by IHD in Alicante. This should prompt the Valencian health authorities to take a close look at this issue, which would surely go unnoticed with a traditional sSMRs-based small area study.

### Case study 3: smoothed life expectancies

Finally, in this third case study, age–space PoDs have been applied to the study of LEs, for each sex, once again for the whole Comunitat Valenciana (CV) for the period 2014–2017. In this case, mortality for all causes has been considered. Mortality data were provided by the mortality registry of the CV. Population data was obtained from the Spanish National Statistics Institute. The spatial aggregation considered was the municipality and 19 age groups: [0, 1), [1, 4], [5, 9), [10, 14),…, [80, 85) and [85,…) were considered for this analysis.

**Fig. 5:**
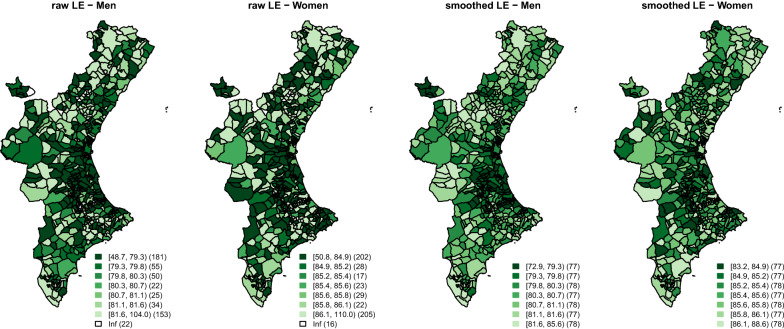
Raw and smoothed life expectancy at birth, for men and women, period 2014–2017

Figure 5 shows the municipal LEs for men and women (separately) for the Valencian municipalities. The two maps on the left side of the figure show the raw LEs, directly calculated from the raw age-specific PoDs. The two maps on the right, show the smoothed counterparts of the maps on the left with the corresponding smoothed LEs. For comparability, cuts for these choropleth maps have been defined according to the smoothed LEs septiles for each sex. The legends of the raw LE plots, highlight the high variability of these indicators. Thus, raw LEs for men range from 48.7 to 104.0 and from 50.8 to 110.0 for women, which seems hard to believe. Moreover, 22 and 16 municipalities, respectively for men and women, showed infinite LEs since no deaths were observed in them during the period of study in the final age interval [8]. Smoothed LEs, however, show much more reasonable (moderate) variability than the raw LEs. This result clearly shows the need for smoothed age-specific PoDs for calculating LEs when dealing with small areas.

Much clearer spatial patterns can be observed in the smoothed LE maps of Fig. 5, compared to their unsmoothed counterparts. Thus, for men, a clear cluster of low LE levels becomes evident in the eastern-central part of CV. This was not so evident according to the raw LEs. Moreover, some areas where the raw LEs showed high heterogeneity (see for example the north-western part of CV), where most of the municipalities are sparsely populated, now show a much more smoothed (spatial) behaviour which seems considerably more reasonable. For women, something similar happens. The smoothed LEs show a clearer spatial pattern and heterogeneity seems to have disappeared for the sparsely populated areas.

Additionally, the smoothed LEs in Fig. 5 allow interesting conclusions to be drawn, such as, for example, that geographical inequalities in men in terms of LEs (12.7 years between the most extreme municipalities) are substantially higher than those in women (5.5 years). According to the raw LEs these differences are reversed, 55.3 years for men and 59.2 years for women, although evidently these values are clearly untenable. The use of smooth LEs allows us to draw some meaningful conclusion in this regard. Finally, we would like to stress that small areas LE analyses provide a complementary view to that provided by sSMR based analyses. LEs take into account not just the mortality at each spatial unit but also the ages when those deaths take place, so LEs analyses complement the results that regular sSMR small areas analyses typically yield.

## Discussion

Although spatio-temporal and multivariate disease mapping analyses on small areas have become quite popular, the same cannot be said for age–space small areas analyses. Nevertheless, as illustrated, the same methods that are used in both spatial and multivariate contexts could also be regularly used for age–space studies where age groups are disaggregated and separately studied, although considering dependence between them.

In this work, the **M**-model is used in order to model the age-specific PoDs for each spatial unit, which assumes stronger dependence between closer age groups and spatial units. As shown, this age–space dependence structure enables information to be transferred between contiguous age groups and neighboring areas, at the same time, providing therefore more reliable (smoothed) age-specific PoDs estimates.

Obviously, there are other alternative possibilities for the age-dependency structure used in the article. However, the one used seems appropriate given that it makes it possible to determine, within the same model, whether or not there is spatial and inter-age group dependency in the data, and even to quantify the strength of these sources of dependency. The small number of age groups in our case studies, and in most of the possible applications that we foresee, could make the discussion and selection of alternative dependency structures for age groups very difficult.

There are multiple reasons to use the **M**-model for age-specific PoDs. The first of them is that it induces both spatial and multivariate dependence by simply multiplying two matrices. Secondly, the **M**-model can be easily generalized to non-separable dependence structures, resulting in more flexible, heteroscedastic, covariance structures [21]. In addition, these models are computationally convenient for modeling multivariate spatial patterns, which makes them an appropriate choice for fitting the data in regular Bayesian inference packages such as WinBUGS, OpenBUGS, Nimble… Moreover, **M**-models have already been implemented for the joint analysis of several correlated spatial patterns in INLA, either for a separable model for a large number of spatial patterns [41], or for an inseparable joint analysis of three spatial patterns [42]. Thus INLA should be also born in mind as a potential tool for this kind of analyses. Finally and the most important in our opinion, is that our proposal facilitates the future incorporation of other factors such as time or sex in a straightforward manner, following the proposal of multidimensional models by Martinez-Beneito et al. [22]. This allows us to consider additional spatial patterns that could be also correlated with the age-specific PoDs that we are modeling in order to enhance the estimates in the model current estimates.

Beyond sASRs and sLEs calculations, the simple study of PoDs for visualizing the different age-specific spatial patterns is also interesting. Moreover, the study of those PoDs is particularly interesting when an inseparable model provides some fit improvement. For example, in another additional case study not presented here, we also applied our proposal for modeling all-cause mortality in the city of Valencia. In that case, applying the **M**-model at space–age group level, the smoothed age specific PoDs showed a pattern with stronger spatial dependence for the youngest age groups (higher values for the corresponding $\gamma _a$) than for the oldest age groups. The corresponding PoDs maps showed as if geographical inequalities existed for the youngest age groups, which pointed towards risk excesses for the deprived neighborhoods, but this effect diluted for older age groups since basically old people die all around while young people die, in general, just in these regions. Therefore, the modeling of non-separable age–space structures seems another additional application of our model, although we have not illustrated with such a detail for questions of space.

In addition, any statistical indicator built on the smoothes age-specific PoDs inherit that smoothed character, yielding therefore more realiable estimates. Thus, in two of the three case studies we have two of the most relevant epidemiological indicators: the age standardized rate and the life expectancy. We have explored their use as an alternative to sSMR for small areas geographical analyses. On one hand, deriving an ASR version of potential use in small areas studies, with the direct comparation posibilities between areas that this brings, should be considered as an additional benefit of our proposal. On the other hand, smoothed life expectancies provides once again a version of this synthetic health indicator of potential use in small areas studies. Studies based on this indicator that take also into account the age of death could bring new possibilities to spatial epidemiological studies. Nevertheless, additional smoothed epidemiological indicators depending on PoDs may also be derived from the smoothed age–space PoDs, such as the number of potential years of life lost. Our study makes possible the study of those indicators also for small areas.

## Supplementary information

**Additional file 1.** This document reproduces the analysis made for all three case studies of the article.
