# Supplementary material for: Beyond standardized mortality ratios; some uses of smoothed age-specific mortality rates on small areas studies
Source: Int J Health Geogr. 2020 Dec 4;19:54. doi: 10.1186/s12942-020-00251-z (PMC7716592; doi:10.1186/s12942-020-00251-z)
Supplement: Supplementary file 1 — Additional file 1. This document reproduces the analysis made for all three case studies of the article. [file 12942_2020_251_MOESM1_ESM.pdf]

Additional file 1.

Beyond standardized mortality ratios; some uses of smoothed age-specific mortality rates on small areas studies.

Perez-Panades J., Botella-Rocamora P. and Martinez-Beneito M.A.

This document reproduces the analysis made at three case studies of the article: "Beyond standardized mortality ratios; some uses of smoothed age-specific mortality rates on small areas studies" by Perez-Panades J., Botella-Rocamora P. and Martinez-Beneito M.A. You can watch the analysis made with full detail at this pdf document.

The statistical analysis below has been run in `R`, by additionally using the library `Rmarkdown`. This document has been executed with real data that are not provided in order to preserve their confidentiality.

Libraries and data loading

```
# Libraries loading
#-----------------
if (!require(RColorBrewer)) {
    install.packages("RColorBrewer")
    library(RColorBrewer)
}
if (!require(rgdal)) {
    install.packages("rgdal")
    library(rgdal)
}
if (!require(sp)) {
    install.packages("sp")
    library(sp)
}
if (!require(spdep)) {
    install.packages("spdep")
    library(spdep)
}
if (!require(Hmisc)) {
    install.packages("Hmisc")
    library(Hmisc)
}
if (!require(R2WinBUGS)) {
    install.packages("R2WinBUGS")
    library(R2WinBUGS)
}
if (!require(epitools)) {
    install.packages("epitools")
    library(epitools)
}
```

```r
if (!require(pbugs)) {
    if (!require(devtools)) {
        install.packages("devtools")
        devtools::install_github("fisabio/pbugs")
    } else {
        install_github("fisabio/pbugs")
    }
}
```

Models

```r
# Mmodel Space-AgeGroup Fully Separable
# %%%%%%%%%%%%%%%%%%%%%%%%%%%%%%%%%%%%%%%%%%%%%%%%%%%%%%%%%%%%%%%%%%%%%%%%%%%%%%%%

MmodelSA <- function() {

    for (a in 1:(nGroups[2])) {
        # AgeGroup Space
        for (s in 1:(nGroups[1])) {
            Obs[s, a] ~ dbin(P[s, a], N[s, a])
            logit(P[s, a]) <- alpha[a] + S12[s, a]
            S12[s, a] <- inprod2(tS1[, s], structure2[, a])
        }
        alpha[a] ~ dflat()
        tS1[a, 1:(nGroups[1])] ~ car.proper(ceros[], C[], adj[], num[],
            M[], prec, gamma)
    }

    for (s in 1:(nGroups[1])) {
        ceros[s] <- 0
    }

    gamma.inf <- min.bound(C[], adj[], num[], M[])
    gamma.sup <- max.bound(C[], adj[], num[], M[])

    gamma ~ dunif(gamma.inf, gamma.sup)

    # Definition structure2 -> AgeGroup (Cholesky Matrix for autoregressive
    # process),
    for (pc in 1:(nGroups[2])) {
        # First row
        structure2[1, pc] <- pow(ro2, pc - 1)
        # Rest of rows
        for (pr in 2:(nGroups[2])) {
            structure2[pr, pc] <- step(pc - pr) * pow(ro2, pc - pr) * pow((1 -
                ro2 * ro2), 0.5)
        }
    }
    ro2 ~ dunif(-1, 1)

    prec <- pow(sdstruct, -2)
    sdstruct ~ dunif(0, 10)
```

```
}


# Mmodel Space-AgeGroup Fully Separable with no interaction
# %%%%%%%%%%%%%%%%%%%%%%%%%%%%%%%%%%%%%%%%%%%%%%%%%%%%%%%%%%%%%%%%%%%%%%%%%%%%%%%%%%

MmodelSA_NoInt <- function() {

    for (a in 1:(nGroups[2])) {
        # AgeGroup Space
        for (s in 1:(nGroups[1])) {
            Obs[s, a] ~ dbin(P[s, a], N[s, a])
            logit(P[s, a]) <- alpha[a] + tS1[s]
        }
        alpha[a] ~ dflat()
    }

    tS1[1:(nGroups[1])] ~ car.proper(ceros[], C[], adj[], num[], M[], prec,
        gamma)

    for (s in 1:(nGroups[1])) {
        ceros[s] <- 0
    }

    gamma.inf <- min.bound(C[], adj[], num[], M[])
    gamma.sup <- max.bound(C[], adj[], num[], M[])

    gamma ~ dunif(gamma.inf, gamma.sup)

    prec <- pow(sdstruct, -2)
    sdstruct ~ dunif(0, 10)
}


# Besag, York and Mollie model
# %%%%%%%%%%%%%%%%%%%%%%%%%%%%%%%%%%%%%%%%%%%%%%%%%%%%%%%%%%%%%%%%%%%%%%%%%%%%%%%%%%

ModeloBYMCarProper <- function() {

    for (i in 1:nreg) {
        O[i] ~ dpois(lambda[i])
        log(lambda[i]) <- log(E[i]) + inter + esp[i]
        RME[i] <- exp(inter + esp[i]) * 100
        probr[i] <- step(inter + esp[i])
    }
    inter ~ dnorm(0, 0.01)
    esp[1:nreg] ~ car.proper(mu.sp[], C[], adj[], num[], M[], tau, rho)
    for (i in 1:nreg) {
        mu.sp[i] <- mu
    }
    rho ~ dunif(rho.low, rho.up)
    rho.low <- min.bound(C[], adj[], num[], M[])
    rho.up <- max.bound(C[], adj[], num[], M[])
```

```
    tau <- pow(sdesp, -2)
    sdesp ~ dunif(0, 2)
    mu ~ dflat()
}
```

Case study 1: interaction between age-groups and space.

Data and call WinBUGS

```
# Lung cancer mortality in women in Comunitat Valenciana (Spain).
# Period 2008-2017 Spatial units of study: 542 municipalities (whole
# Comunitat Valenciana, CV) Age: 11 age intervals.

# Mean population Comunitat Valenciana 2008-2017 Women 11AgeGroups
load(file = "Data/MeanPop_Women_11AgeGroups_2008-2017_CV.RData")
# Mortality Lung Comunitat Valenciana 2008-2017 Women 11AgeGroups
load(file = "Data/Mortality_LungCancer_Women_11AgeGroups_2008-2017_CV.RData")

dim(mort)
dim(pob)
# [1] 542 11 [1] 542 11

# There are a total of 4,232 deaths for the whole period of study.
sum(mort)
# 4232

# Data set has 4,646 cells out of the 5,962 municipality-age groups
# combinations (78%) with zero deaths
sum(mort == 0)
# 4656

# Age groups of municipalities whose population is zero in the study
# period are replaced by 1 for correct modelling with binomial
# distribution.
pob[pob == 0] <- 1


# Preparing and running models
nGroups <- dim(mort)[1:2]  #SC,GEdad = 531 12
NSC <- dim(mort)[1]
NGed <- dim(mort)[2]

# Cartography
carto <- rgdal::readOGR(dsn = "Data/Carto", layer = "mapa_cv_muni", verbose = FALSE)
sp::proj4string(carto) <- sp::CRS("+proj=longlat +datum=WGS84")
index <- order(carto$INE_MUN)
carto <- carto[index, ]

# Neighborhood structure is created for the CAR.normal distribution.
Veci <- spdep::poly2nb(carto)
```

```r
# Neighbourhood relations are added to join an 'island' of the
# cartography of the Valencian Community to the municipalities closest
# to it.
Veci[[317]] <- as.integer(c(Veci[[317]], 364))
Veci[[364]] <- as.integer(c(Veci[[364]], 317))
Veci[[477]] <- as.integer(c(Veci[[477]], 312))
Veci[[312]] <- as.integer(c(Veci[[312]], 477))

# Convert neighborhood structure to Winbugs
VeciWB <- spdep::nb2WB(Veci)


# WinBUGS call for Mmodel SA with interaction
# %%%%%%%%%%%%%%%%%%%%%%%%%%%%%%%%%%%%%%%%%%%%%%%%%%%%%%%%%%%%%%%%%%%%%%%%%%%%%%%%
datos <- list(Obs = mort[, ], N = pob[, ], nGroups = nGroups, adj = VeciWB$adj,
    num = VeciWB$num, C = rep(1/VeciWB$num, VeciWB$num), M = 1/VeciWB$num)

set.seed(1)

# alpha is initialized into the mean values by age group to accelerate
# model convergence.
medias <- matrix(as.numeric(round(logit(apply(mort/pob, 2, mean)), 0)),
    nrow = nGroups[2], ncol = 1, byrow = F)
iniciales <- function() {
    list(alpha = rnorm(nGroups[2], medias, 0.5), gamma = runif(1, 0.4,
        0.6), ro2 = runif(1, 0.5, 1), sdstruct = runif(1, 0.2, 0.5), tS1 = array(rnorm(nGroups[1] *
        nGroups[2], 0, 1), dim = c(nGroups[2], nGroups[1])))
}

param <- c("P", "alpha", "gamma", "ro2", "sdstruct")

niters <- 30000
nburns <- 5000

resul <- list()

resul$tiempo <- system.time(resul$WinBUGS <- pbugs(data = datos, inits = iniciales,
    parameters.to.save = param, model = MmodelSA, n.iter = niters, n.burnin = nburns,
    n.chains = 3, DIC = F, pbugs.directory = path.expand("~/.wine/dosdevices/c:"),
    debug = F, bugs.seed = 1))


save(resul, file = "Results/Res_LungWomen_0817_CV_MmodelSA_30000it.Rdata")


# WinBUGS call for Mmodel SA with NO interaction
# %%%%%%%%%%%%%%%%%%%%%%%%%%%%%%%%%%%%%%%%%%%%%%%%%%%%%%%%%%%%%%%%%%%%%%%%%%%%%%%%

datos <- list(Obs = mort[, ], N = pob[, ], nGroups = nGroups, adj = VeciWB$adj,
    num = VeciWB$num, C = rep(1/VeciWB$num, VeciWB$num), M = 1/VeciWB$num)

set.seed(1)
```

```r
# alpha is initialized into the mean values by age group to accelerate
# model convergence.
medias <- matrix(as.numeric(round(logit(apply(mort/pob, 2, mean)), 0)),
    nrow = nGroups[2], ncol = 1, byrow = F)
iniciales <- function() {
    list(alpha = rnorm(nGroups[2], medias, 0.5), gamma = runif(1, 0.4,
        0.6), sdstruct = runif(1, 0.2, 0.5), tS1 = rnorm(nGroups[1], 0,
        1))
}

param <- c("P", "alpha", "gamma", "sdstruct")

niters <- 30000
nburns <- 5000

resul <- list()

resul$tiempo <- system.time(resul$WinBUGS <- pbugs(data = datos, inits = iniciales,
    parameters.to.save = param, model = modelo_seleccionado, n.iter = niters,
    n.burnin = nburns, n.chains = 3, DIC = F, pbugs.directory = path.expand("~/.wine/dosdevices/c:"),
    debug = F, bugs.seed = 1))


save(resul, file = "Data/Res_LungWomen_0817_CV_MmodelSA_noInt_30000it.Rdata")
```

Results: Convergence checking Mmodel SA

```r
# Load Population
load("Data/MeanPop_Women_11AgeGroups_2008-2017_CV.RData")
pob[pob == 0] <- 1
# Load mortality
load("Data/Mortality_LungCancer_Women_11AgeGroups_2008-2017_CV.RData")
# Load results
load("Results/Res_LungWomen_0817_CV_modelSA_30000it.Rdata")
ResulModel <- resul$WinBUGS
rm(resul)
# Load cartography
carto <- rgdal::readOGR(dsn = "Data/Carto", layer = "mapa_cv_muni", verbose = FALSE)
proj4string(carto) <- sp::CRS("+proj=longlat +datum=WGS84")
index <- order(carto$INE_MUN)
carto <- carto[index, ]

# TT are the number of years in the period of study.
TT <- 10

# convergence checking
p <- ResulModel$summary[is.element(substr(row.names(ResulModel$summary),
    1, 1), c("P")), ]
p1 <- p[order(p[, 8], decreasing = T), ][1:3, ]
p1 <- cbind(apply(p1[, 1:8], 2, round, digits = 3), p1[, 9])
colnames(p1)[9] <- "n.eff"
p2 <- p[order(p[, 9], decreasing = F), ][1:3, ]
```

```r
p2 <- cbind(apply(p2[, 1:8], 2, round, digits = 3), p2[, 9])
colnames(p2)[9] <- "n.eff"
others.p <- as.data.frame.matrix(ResulModel$summary[!is.element(substr(row.names(ResulModel$summary),
    1, 1), c("P")), ])
others.p <- cbind(apply(others.p[, 1:8], 2, round, digits = 3), others.p[,
    9])
colnames(others.p)[9] <- "n.eff"
```

The Brooks-Gelman-Rubin statistic and the effective sample size are checked for all model parameters (5962 probabilities estimates, 542 municipalities x 11 age groups). Below are those estimates with higher values of Rhat or lower effective size.

	mean	sd	2.5%	25%	50%	75%	97.5%	Rhat	n.eff
P[157,7]	0.003	0.001	0.002	0.002	0.003	0.003	0.004	1.015	140
P[298,8]	0.004	0.001	0.003	0.003	0.004	0.004	0.006	1.015	140
P[229,7]	0.003	0.001	0.002	0.002	0.003	0.003	0.004	1.014	140

	mean	sd	2.5%	25%	50%	75%	97.5%	Rhat	n.eff
P[157,7]	0.003	0.001	0.002	0.002	0.003	0.003	0.004	1.015	140
P[197,7]	0.003	0.001	0.001	0.002	0.003	0.003	0.005	1.014	140
P[229,7]	0.003	0.001	0.002	0.002	0.003	0.003	0.004	1.014	140

For the rest of the parameters we get:

	mean	sd	2.5%	25%	50%	75%	97.5%	Rhat	n.eff
alpha[1]	-10.284	0.198	-10.670	-10.410	-10.280	-10.160	-9.889	1.002	1000
alpha[2]	-7.715	0.167	-8.065	-7.819	-7.716	-7.607	-7.390	1.012	300
alpha[3]	-6.803	0.148	-7.120	-6.894	-6.801	-6.713	-6.497	1.017	240
alpha[4]	-6.232	0.150	-6.548	-6.323	-6.223	-6.146	-5.920	1.013	220
alpha[5]	-5.847	0.148	-6.177	-5.927	-5.843	-5.761	-5.554	1.023	340
alpha[6]	-5.839	0.143	-6.157	-5.913	-5.840	-5.761	-5.542	1.017	140
alpha[7]	-5.741	0.151	-6.062	-5.820	-5.736	-5.652	-5.451	1.021	130
alpha[8]	-5.668	0.140	-5.960	-5.755	-5.662	-5.580	-5.400	1.020	120
alpha[9]	-5.438	0.141	-5.739	-5.524	-5.434	-5.345	-5.189	1.022	160
alpha[10]	-5.403	0.142	-5.708	-5.486	-5.398	-5.314	-5.131	1.011	300
alpha[11]	-5.261	0.144	-5.571	-5.341	-5.253	-5.178	-4.956	1.016	150
gamma	0.991	0.009	0.969	0.989	0.994	0.997	0.999	1.030	80
ro2	0.875	0.047	0.769	0.846	0.878	0.909	0.950	1.007	320
sdstruct	0.488	0.054	0.389	0.450	0.486	0.523	0.601	1.020	140

Results: Convergence checking Mmodel with no interaction

```r
# Load Population
load("Data/MeanPop_Women_11AgeGroups_2008-2017_CV.RData")
pob[pob == 0] <- 1
# Load mortality
load("Data/Mortality_LungCancer_Women_11AgeGroups_2008-2017_CV.RData")
# Load results
```

```r
load("Results/Res_LungWomen_0817_CV_modelSA_NoInt_30000it.Rdata")
ResulModelNoInt <- resul$WinBUGS
rm(resul)

# convergence checking
p <- ResulModelNoInt$summary[is.element(substr(row.names(ResulModelNoInt$summary),
    1, 1), c("P")), ]
p1 <- p[order(p[, 8], decreasing = T), ][1:3, ]
p1 <- cbind(apply(p1[, 1:8], 2, round, digits = 3), p1[, 9])
colnames(p1)[9] <- "n.eff"
p2 <- p[order(p[, 9], decreasing = F), ][1:3, ]
p2 <- cbind(apply(p2[, 1:8], 2, round, digits = 3), p2[, 9])
colnames(p2)[9] <- "n.eff"

others.p <- as.data.frame.matrix(ResulModelNoInt$summary[!is.element(substr(row.names(ResulModelNoInt$su
    1, 1), c("P")), ])
others.p <- cbind(apply(others.p[, 1:8], 2, round, digits = 3), others.p[,
    9])
colnames(others.p)[9] <- "n.eff"
```

The Brooks-Gelman-Rubin statistic and the effective sample size are checked for all model parameters (5962 probabilities estimates, 542 municipalities x 11 age groups). Below are those estimates with higher values of Rhat or lower effective size.

	mean	sd	2.5%	25%	50%	75%	97.5%	Rhat	n.eff
P[140,8]	0.005	0.001	0.003	0.004	0.004	0.005	0.007	1.013	210
P[140,3]	0.001	0.000	0.001	0.001	0.001	0.002	0.002	1.012	200
P[433,2]	0.000	0.000	0.000	0.000	0.000	0.001	0.001	1.012	240

	mean	sd	2.5%	25%	50%	75%	97.5%	Rhat	n.eff
P[10,2]	0.000	0.000	0.000	0.000	0.000	0.001	0.001	1.011	180
P[94,11]	0.008	0.002	0.005	0.007	0.008	0.009	0.011	1.011	180
P[140,2]	0.001	0.000	0.000	0.000	0.001	0.001	0.001	1.011	180

For the rest of the parameters we get:

	mean	sd	2.5%	25%	50%	75%	97.5%	Rhat	n.eff
alpha[1]	-10.305	0.176	-10.650	-10.420	-10.300	-10.190	-9.965	1.005	400
alpha[2]	-7.731	0.134	-7.996	-7.813	-7.736	-7.649	-7.444	1.001	1000
alpha[3]	-6.793	0.115	-7.015	-6.861	-6.798	-6.729	-6.551	1.015	190
alpha[4]	-6.210	0.110	-6.420	-6.277	-6.214	-6.153	-5.974	1.012	220
alpha[5]	-5.816	0.107	-6.032	-5.879	-5.819	-5.759	-5.591	1.009	350
alpha[6]	-5.796	0.109	-6.000	-5.861	-5.799	-5.743	-5.567	1.009	270
alpha[7]	-5.665	0.107	-5.867	-5.727	-5.668	-5.606	-5.429	1.011	320
alpha[8]	-5.630	0.107	-5.838	-5.689	-5.631	-5.573	-5.403	1.008	500
alpha[9]	-5.427	0.109	-5.634	-5.485	-5.431	-5.373	-5.187	1.012	240
alpha[10]	-5.410	0.109	-5.622	-5.474	-5.415	-5.350	-5.190	1.006	540
alpha[11]	-5.263	0.108	-5.462	-5.326	-5.268	-5.204	-5.031	1.014	220
gamma	0.976	0.035	0.892	0.971	0.986	0.993	0.999	1.028	660
sdstruct	0.446	0.060	0.341	0.403	0.442	0.484	0.570	1.000	1000

Results: DIC comparison between models with and without interaction

```r
# DIC.LogisticReg FUNCTION
# %%%%%%%%%%%%%%%%%%%%%%%%%%%%%%%%%%%%%%%%%%%%%%%%%%%%%%%%%%%%%%%%%%%%%%%%%%%%%%%%

DIC.LogisticReg <- function(ys, Psim, ns) {
    # DevBin = Deviance for Binomial data, y~dbin(p,n)
    DevBin <- function(y, p, n) {
        -2 * (sum(y * log(p)) + sum((n - y) * log(1 - p)) + sum(lchoose(n,
            y)))
    }
    # DPM = Deviance of posterior mean of P
    DPM <- DevBin(ys, apply(Psim, c(2, 3), mean), ns)
    # PMD = Posterior mean deviance
    PMD <- mean(apply(Psim, 1, DevBin, y = mort, n = pob))
    # pD = Effective number of parameters
    pD <- PMD - DPM
    # DIC = Deviance Information Criterion
    DIC <- PMD + pD
    return(DIC)
}

# DIC comparison %%%%%%%%%%%%%%%

# Mmodel SA
DIC.LogisticReg(mort, ResulModel$sims.list$P, pob)
```

```
## [1] 6507.982
```

```r
# Mmodel SA with no interaction
DIC.LogisticReg(mort, ResulModelNoInt$sims.list$P, pob)
```

```
## [1] 6546.502
```

Results: Decomposition of logit(PoDs) variability

```r
# Logit function
# %%%%%%%%%%%%%%%%%%%%%%%%%%%%%%%%%%%%%%%%%%%%%%%%%%%%%%%%%%%%%%%%%%%%%%%%%%%%%%%%
logit <- function(p) {
    log(p/(1 - p))
}

AgeGroupLungLab <- c("<40", "40-44", "45-49", "50-54", "55-59", "60-64",
    "65-69", "70-74", "75-79", "80-84", ">=85")

# Mean estimated probabilities
sP <- ResulModel$mean$P  #dim 542 x 11
# We apply the logit function to the probabilities
logitsP <- logit(sP)

# A posteriori distribution of the probabilities of death in each age
# group and municipality
sP.sim <- ResulModel$sims.matrix[, which(substr(dimnames(ResulModel$sims.matrix)[[2]],
```

```r
    1, 1) == "P")]
# dim(sP.sim) 1002 5962 # = 542 munis x 11 GrEdad We apply the logit
# function to the probabilities
logitsP.sim <- logit(sP.sim)

# Spatial component
sPmedioByMuni <- apply(logitsP, 1, mean)
Scomp <- sPmedioByMuni - mean(logitsP)

# Age group component
sPmedioByPeriodo <- apply(logitsP, 2, mean)
Tcomp <- sPmedioByPeriodo - mean(logitsP)
Tcomp.sim <- t(apply(logitsP.sim, 1, function(x) {
    y <- matrix(x, ncol = ncol(logitsP), byrow = T)
    apply(y, 2, mean) - mean(y)
}))
Tcomp.l <- apply(Tcomp.sim, 2, quantile, 0.025)
Tcomp.u <- apply(Tcomp.sim, 2, quantile, 0.975)

# Interaction component
STcomp <- logitsP - (outer(Scomp, Tcomp, FUN = "+") + mean(logitsP))




# FIG1. raw vs smoothed, PoDs
# %%%%%%%%%%%%%%%%%%%%%%%%%%%%%%%%%%%%%%%%%%%%%%%%%%%%%%%%%%%%%%%%%%%%%%%%%%%%%%

# Raw PoDs
rawP <- mort/(pob)
# Smoothed PoDs
smoP <- sP

rawP.df <- apply(mort, 2, sum)/apply(pob, 2, sum)
rawP.df <- rbind(rawP.df, rawP[which(row.names(rawP) == "12009"), ])
rawP.df <- rbind(rawP.df, rawP[which(row.names(rawP) == "03063"), ])
row.names(rawP.df) <- c("CV", "Almazora", "Denia")

smoP.df <- apply(sP, 2, mean)
smoP.df <- rbind(smoP.df, smoP[which(row.names(rawP) == "12009"), ])
smoP.df <- rbind(smoP.df, smoP[which(row.names(rawP) == "03063"), ])
row.names(smoP.df) <- c("CV", "Almazora", "Denia")
```

```r
# postscript('../Articulo_Mmodelv4/FigurasASR/rawPoDs_vs_sPoDs_todosperfiles_Mmodel_logitsP_PoDs.eps',
# width=12, height=5)
par(mfrow = c(1, 2), mar = c(2, 4, 2, 3))
plot(1:11, rawP[1, ], type = "l", ylim = c(0, 0.005 * TT), col = grey(0.89),
    xaxt = "n", xlab = "Age groups", ylab = "raw PoDs", bty = "l")
axis(1, at = 1:11, labels = AgeGroupLungLab, cex.axis = 0.5)
for (i in 2:542) {
    lines(1:11, rawP[i, ], type = "l", col = grey(0.89))
}
lines(1:11, rawP.df[1, ], type = "o", col = "olivedrab", pch = 16, lty = 1)
lines(1:11, rawP.df[2, ], type = "o", pch = 17, lty = 1, col = "blue")
```

```r
lines(1:11, rawP.df[3, ], type = "o", pch = 15, lty = 1, col = "sienna")
legend(1.5, 0.005 * TT, legend = c("Denia", "CV", "Almazora"), lty = c(1,
    1, 1), pch = c(15, 16, 17), col = c("sienna", "olivedrab", "blue"),
    bg = "white")

plot(1:11, smoP[1, ], type = "l", ylim = c(0, 0.005 * TT), col = grey(0.89),
    xaxt = "n", xlab = "Age groups", ylab = "smoothed PoDs", bty = "l")
axis(1, at = 1:11, labels = AgeGroupLungLab, cex.axis = 0.5)
for (i in 2:542) {
    lines(1:11, smoP[i, ], type = "l", col = grey(0.89))
}
lines(1:11, smoP.df[1, ], type = "o", col = "olivedrab", pch = 16, lty = 1)
lines(1:11, smoP.df[2, ], type = "o", pch = 17, lty = 1, col = "blue")
lines(1:11, smoP.df[3, ], type = "o", pch = 15, lty = 1, col = "sienna")
legend(1.5, 0.005 * TT, legend = c("Denia", "CV", "Almazora"), lty = c(1,
    1, 1), pch = c(15, 16, 17), col = c("sienna", "olivedrab", "blue"),
    bg = "white")
```

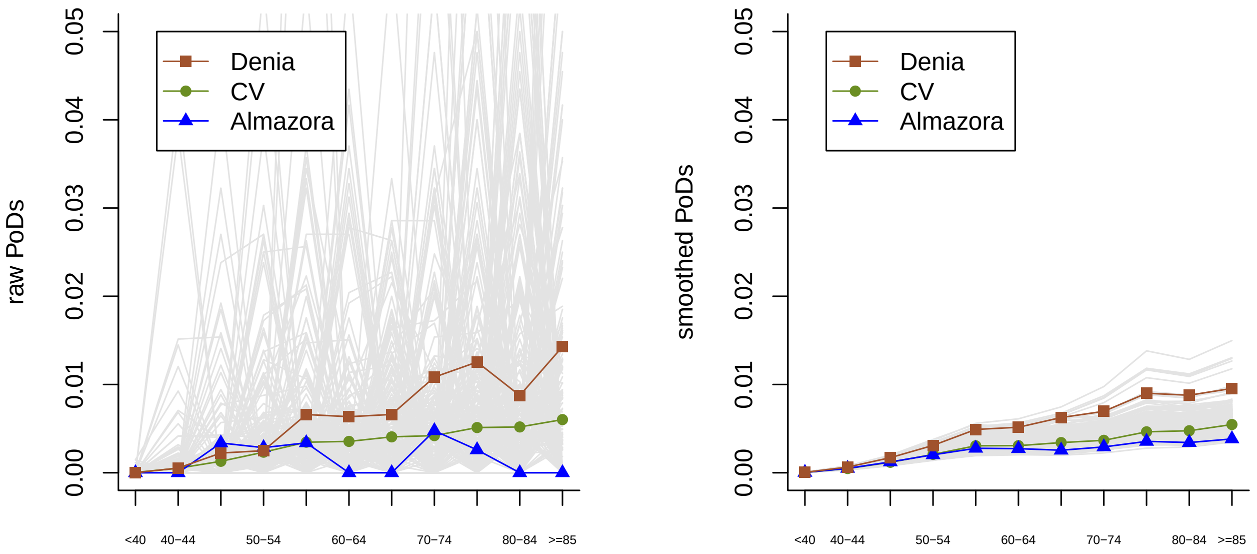


```r
# dev.off()
```

FIG1.Comparison between raw and smoothed PoDs from lung cancer in women in 2008-2017.

The age effect unsurprisingly shows an upwards trend of the risks as a function of age:

```r
par(mar = c(4, 4, 2, 1), cex.axis = 0.8, cex.main = 1, cex.lab = 0.9)
plot(1:ncol(logitsP), Tcomp, type = "l", main = "Age group effect", xaxt = "n",
    xlab = "Age group", ylab = "logit(P*)")
axis(1, at = 1:ncol(logitsP), labels = AgeGroupLungLab)
lines(1:ncol(logitsP), Tcomp.l, lty = 2)
lines(1:ncol(logitsP), Tcomp.u, lty = 2)
abline(h = 0, lty = 3)
```

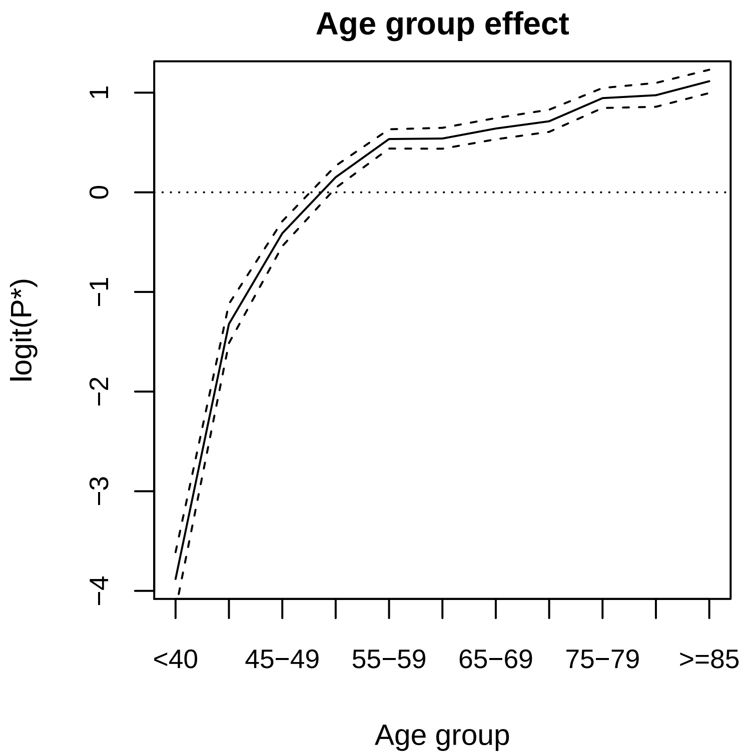

Figure 2 shows the spatial term, at the left hand side plot, a clear spatial pattern, with high risks mainly at the southern side of CV. The right hand side plot shows the interaction term for each municipality (one line per municipality):

```
# Space Component and interaction EPS
# %%%%%%%%%%%%%%%%%%%%%%%%%%%%%%%%%%%%%%%%%%%%%%%%%%%%%%%%%%%%%%%%%%%%%%%%%%%%%%

# postscript('../Articulo_Mmodelv4/FigurasASR/SpaceAndInteraction_gris_MMultDim_logitsP_PoDs.eps',
# width=12, height=5)
par(mfrow = c(1, 2), mar = c(2, 4, 2, 3), cex.axis = 0.6, cex.main = 1,
    cex.lab = 0.9)
# Representaci?n Efecto Espacial Medio
Cortes <- cut2(Scomp, cuts = quantile(Scomp, seq(0, 1, 1/7)))
valores <- as.numeric(Cortes)
mypalette <- brewer.pal(7, "BrBG")
mipaleta <- mypalette[length(mypalette):1]
fgs <- mipaleta[as.vector(valores)]
plot(carto, col = fgs, xlab = "", ylab = "", axes = F, main = "Spatial effect")
levelsCortes <- c("[-0.38,-0.16)", "[-0.16,-0.08)", "[-0.08,-0.04)", "[-0.04,0)",
    "[0, 0.05)", "[ 0.05, 0.16)", "[ 0.16, 0.75]")
auxgrafcompesp <- matrix(c(levelsCortes, paste("(", as.vector(table(Cortes)),
    ")", sep = "")), nrow = 7, ncol = 2)
leyenda <- apply(auxgrafcompesp, 1, paste, collapse = " ")
legend("bottomright", legend = leyenda, fill = mipaleta, border = mipaleta,
    cex = 0.6, title = paste0("logit(P*)"), bty = "n")

# Evoluciones del efecto espacio-temporal por Municipio
plot(1:ncol(sP), STcomp[1, ], type = "l", ylim = c(-0.4, 0.4), ylab = "",
    main = "Space x Age group interaction", xaxt = "n", col = grey(0.89))
axis(1, at = 1:ncol(sP), labels = AgeGroupLungLab, crt = 45)
```

```
for (cont in 2:nrow(sP)) {
    lines(1:ncol(sP), STcomp[cont, ], col = grey(0.89))
}
# Buscamos aquellos munis con maxima y m?nima evoluci?n (lineal)
lines(1:ncol(sP), STcomp[which(row.names(pob) == "12009"), ], col = "blue",
    lwd = 2)  # 12009 = Almazora
lines(1:ncol(sP), STcomp[which(row.names(pob) == "03063"), ], col = "sienna",
    lwd = 2)  # 03063 = Denia
legend("topleft", legend = c("Almazora", "Denia"), col = c("blue", "sienna"),
    lty = 1, lwd = 2, bty = "n")
abline(h = 0, lty = 3, col = gray(0.7))
```

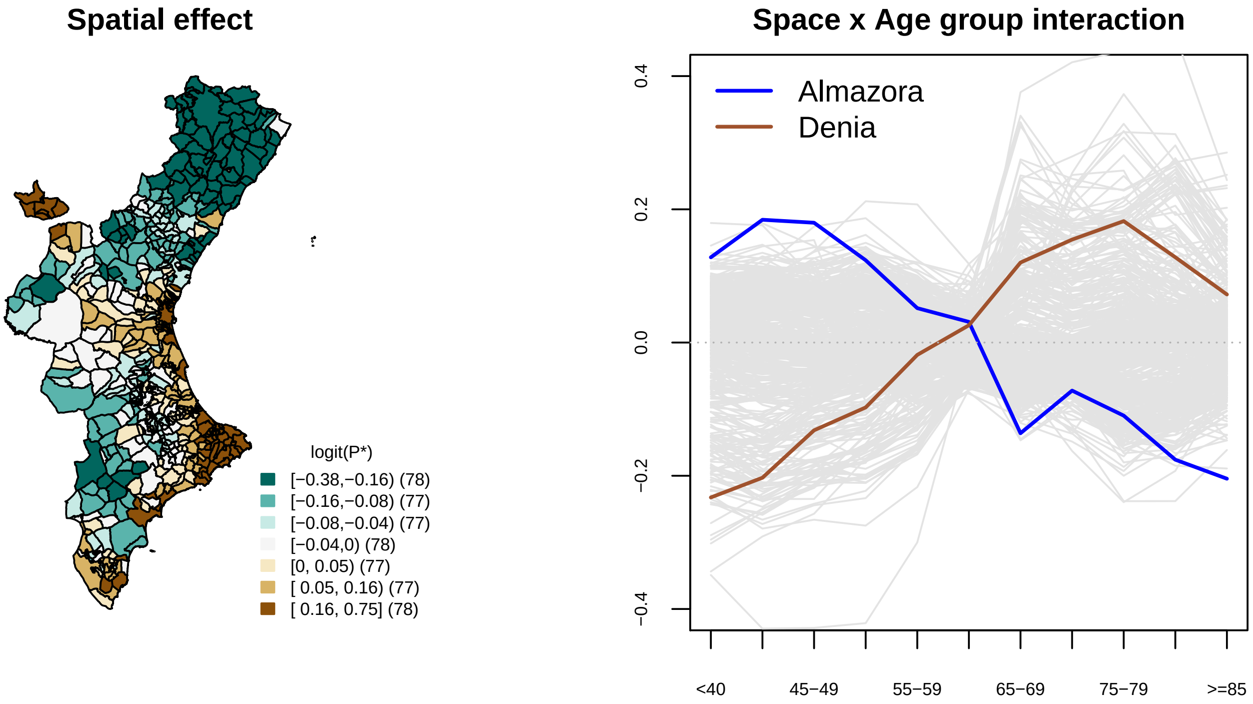


```
# dev.off()
```

FIG2. Spatial and interaction terms for the smoothed age-specific PoDs.

Figure 3 shows choropleth maps for the interaction term for several of the age groups considered. Almazora and Denia have been highlighted in the first of these plots.

```
# postscript('../Articulo_Mmodelv4/FigurasASR/Space_AgeGroup_interaction_titulo2_almdenia_MMultDim_1x4_
# width=9, height=4)
par(mfrow = c(1, 4), mar = c(1, 1, 1, 1), cex.axis = 0.6, cex.main = 0.9,
    cex.lab = 0.6)
for (i in c(1, 5, 8, 11)) {
    STcm <- STcomp
    auxquantile <- apply(STcm, 2, quantile, prob = seq(0, 1, 1/7))
    CortesFac <- Hmisc::cut2(STcm[, i], cuts = c(min(auxquantile), apply(auxquantile[2:7,
        ], 1, mean), max(auxquantile)))
    valores <- as.numeric(CortesFac)
    mypalette <- RColorBrewer::brewer.pal(7, "BrBG")
    mipaleta <- mypalette[length(mypalette):1]
```

```r
    fgs <- mipaleta[valores]
    plot(carto, col = fgs, xlab = "", ylab = "", axes = F)
    if (i == 1) {
        text(0.5, 40, "Almazora")
        segments(x0 = 0, y0 = 39.93, x1 = 0.17, y1 = 40)
        text(0.4, 39, "Denia")
        segments(x0 = 0.1, y0 = 38.85, x1 = 0.17, y1 = 39)
    }
    auxgrafcompesp <- matrix(c("[-0.43,-0.08)", "[-0.08,-0.04)", "[-0.04,-0.01)",
        "[-0.01, 0.01)", "[ 0.01, 0.04)", "[ 0.04, 0.08)", "[ 0.08, 0.46]",
        paste("(", as.numeric(table(factor(valores - 1, levels = 0:6, labels = 0:6))),
            ")", sep = "")), nrow = 7, ncol = 2)
    legend("bottomright", legend = apply(auxgrafcompesp, 1, paste, collapse = " "),
        fill = mipaleta, border = mipaleta, cex = 0.6, title = paste0("interac. comp."),
        bty = "n")
    title(paste("Age Group ", AgeGroupLungLab[i], sep = ""), cex.main = 0.9)
}
```

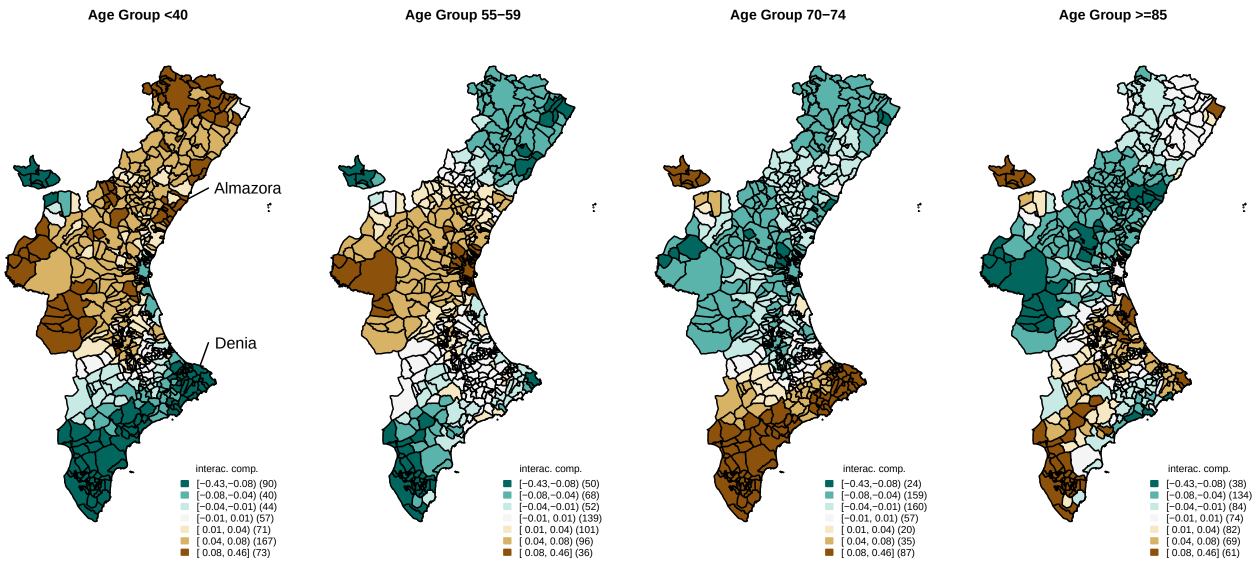


```r
# dev.off()
```

Fig 3. Age-space group interaction component.

Correlation of the spatial component and the interaction terms:

```r
corSpaInt <- round(apply(STcomp, 2, function(v, Scomp) {
    cor(Scomp, v)
}, Scomp), 2)
names(corSpaInt) <- AgeGroupLungLab
```

<40	40-44	45-49	50-54	55-59	60-64	65-69	70-74	75-79	80-84	>=85
-0.65	-0.53	-0.42	-0.14	0.14	0.37	0.42	0.42	0.25	0.18	0.32

Case study 2: smoothed ASRs

Running the models

```
# Data Valencia
# %%%%%%%%%%%%%%%%%%%%%%%%%%%%%%%%%%%%%%%%%%%%%%%%%%%%%%%%%%%%%%%%%%%%%%%%%%%%%%

# Ischaemic mortality in women in Valencia, Comunitat Valenciana
# (Spain).  Period 1996-2015 Spatial units of study: 531 census tracts
# (whole Valencia city, Comunitat Valenciana, Spain) Age: 9 age
# intervals.

# Mean population Valencia 1996-2015 Women 9AgeGroups rounded zero
# decimals
load(file = "Data/MeanPop_Women_9AgeGroups_1996-2015_VAL.RData")
# Mortality Ischaemic Valencia 1996-2015 Women 9AgeGroup
load(file = "Data/Mortality_Ischaemic_Women_9AgeGroups_1996-2015_VAL.RData")

dim(mort)
dim(pob)
# [1] 531 9 [1] 531 9

# There are a total of 8,319 deaths for the whole period of study.
sum(mort)
# 6434

# Lines 644:653 are not necessary here.  Age groups of municipalities
# whose population is zero in the study period are replaced by 1 for
# correct modelling with binomial distribution. pob[pob==0]<-1

# We observe 7 combinations AgeGroup-Areas in which the mortality is
# higher than its mean population mort[mort>pob] pob[mort>pob] In those
# AgeGroup-Areas, we change the population for the mortality
# pob[mort>pob] <- mort[mort>pob]


# Expected and Observed values by census tract for BYM model
Expected <- pob %*% (apply(mort, 2, sum)/apply(pob, 2, sum))
Observed <- apply(mort, 1, sum)
# Format
Expected <- as.vector(Expected)
names(Expected) <- names(Observed)




# Preparing and running the Mmodel SA for Valencia
# %%%%%%%%%%%%%%%%%%%%%%%%%%%%%%%%%%%%%%%%%%%%%%%%%%%%%%%%%%%%%%%%%%%%%%%%%%%%%%

nGroups <- dim(mort)[1:2]  #SC,GEdad = 531 9
NSC <- dim(mort)[1]
NGed <- dim(mort)[2]

# Cartography
```

```r
load("Data/Carto/carto_valencia.RData")
Veci <- spdep::poly2nb(carto.valencia)
# Convert neighborhood structure to Winbugs
VeciWB <- spdep::nb2WB(Veci)

datos <- list(Obs = mort[, ], N = pob[, ], nGroups = nGroups, adj = VeciWB$adj,
    num = VeciWB$num, C = rep(1/VeciWB$num, VeciWB$num), M = 1/VeciWB$num)

set.seed(1)

# alpha is initialized into the mean values by age group to accelerate
# model convergence.
medias <- matrix(as.numeric(round(logit(apply(mort/pob, 2, mean)), 0)),
    nrow = nGroups[2], ncol = 1, byrow = F)
iniciales <- function() {
    list(alpha = rnorm(nGroups[2], medias, 0.5), gamma = runif(1, 0.4,
        0.6), ro2 = runif(1, 0.5, 1), sdstruct = runif(1, 0.2, 0.5), tS1 = array(rnorm(nGroups[1] *
        nGroups[2], 0, 1), dim = c(nGroups[2], nGroups[1])))
}

param <- c("P", "alpha", "gamma", "ro2", "sdstruct")

niters <- 30000
nburns <- 5000

resul <- list()

resul$tiempo <- system.time(resul$WinBUGS <- pbugs(data = datos, inits = iniciales,
    parameters.to.save = param, model = MmodelSA, n.iter = niters, n.burnin = nburns,
    n.chains = 3, DIC = F, pbugs.directory = path.expand("~/.wine/dosdevices/c:"),
    debug = F, bugs.seed = 1))


save(resul, file = "Data/Res_IsqWomen_9615_VAL_MmodelSA_30000it.Rdata")




# Preparing and running the BYM model for Valencia
# %%%%%%%%%%%%%%%%%%%%%%%%%%%%%%%%%%%%%%%%%%%%%%%%%%%%%%%%%%%%%%%%%%%%%%%%%%%%%%%

set.seed(1)

inits.BYM <- function() {
    list(inter = rnorm(1), esp = rnorm(nrow(mort)), sdesp = runif(1, 0,
        0.2), mu = rnorm(1))
}

datos.BYM <- list(O = Observed, E = Expected, nreg = nrow(mort), num = VeciWB$num,
    adj = VeciWB$adj, M = 1/VeciWB$num, C = rep(1/VeciWB$num, VeciWB$num))


par.BYM <- c("RME", "probr", "mu", "sdesp", "rho")
```

```r
niters <- 30000
nburns <- 5000

resul <- list()


resul$tiempo <- system.time(resul$WinBUGS <- pbugs(data = datos.BYM, inits = inits.BYM,
    parameters.to.save = par.BYM, model = modelo_seleccionado, n.iter = niters,
    n.burnin = nburns, n.chains = 3, DIC = F, pbugs.directory = path.expand("~/.wine/dosdevices/c:"),
    debug = F, bugs.seed = 1))

save(resul, file = paste0("Data/Res_IsqWomen_9615_VAL_ModeloBYMCarProper_30000it.Rdata"))






# Data Alicante
# %%%%%%%%%%%%%%%%%%%%%%%%%%%%%%%%%%%%%%%%%%%%%%%%%%%%%%%%%%%%%%%%%%%%%%%%%%%%%%%%

# Ischaemic mortality in women in Alicante, Comunitat Valenciana
# (Spain).  Period 1996-2015 Spatial units of study: 178 census tracts
# (whole Alicante, Comunitat Valenciana, Spain) Age: 9 age intervals.

# Mean population Valencia 1996-2015 Women 9AgeGroups rounded zero
# decimals
load(file = "Data/MeanPop_Women_9AgeGroups_1996-2015_ALI.RData")
# Mortality Ischaemic Valencia 1996-2015 Women 9AgeGroup
load(file = "Data/Mortality_Ischaemic_Women_9AgeGroups_1996-2015_ALI.RData")

dim(mort)
dim(pob)
# [1] 178 9 [1] 178 9

# There are a total of 3,800 deaths for the whole period of study.
sum(mort)
# 3974

# Age groups of municipalities whose population is zero in the study
# period are replaced by 1 for correct modelling with binomial
# distribution. pob[pob==0]<-1

# We observe 4 combinations AgeGroup-Areas in which the mortality is
# higher than its mean population
mort[mort > pob]
# [1] 25 51 15 16
pob[mort > pob]
# [1] 24 44 9 11

# In those AgeGroup-Areas, we change the population for the mortality
pob[mort > pob] <- mort[mort > pob]
```

```
# Preparing and running the Mmodel SA for Alicante
# %%%%%%%%%%%%%%%%%%%%%%%%%%%%%%%%%%%%%%%%%%%%%%%%%%%%%%%%%%%%%%%%%%%%%%%%%%%%%%

nGroups <- dim(mort)[1:2]  #SC,GEdad = 178 9
NSC <- dim(mort)[1]
NGed <- dim(mort)[2]

# Cartography
load("Data/Carto/carto_alicante.RData")
Veci <- spdep::poly2nb(carto.alicante)
# Convert neighborhood structure to Winbugs
VeciWB <- spdep::nb2WB(Veci)

datos <- list(Obs = mort[, ], N = pob[, ], nGroups = nGroups, adj = VeciWB$adj,
    num = VeciWB$num, C = rep(1/VeciWB$num, VeciWB$num), M = 1/VeciWB$num)

set.seed(1)

# alpha is initialized into the mean values by age group to accelerate
# model convergence.
medias <- matrix(as.numeric(round(logit(apply(mort/pob, 2, mean)), 0)),
    nrow = nGroups[2], ncol = 1, byrow = F)
iniciales <- function() {
    list(alpha = rnorm(nGroups[2], medias, 0.5), gamma = runif(1, 0.4,
        0.6), ro2 = runif(1, 0.5, 1), sdstruct = runif(1, 0.2, 0.5), tS1 = array(rnorm(nGroups[1] *
        nGroups[2], 0, 1), dim = c(nGroups[2], nGroups[1])))
}

param <- c("P", "alpha", "gamma", "ro2", "sdstruct")

niters <- 30000
nburns <- 5000

resul <- list()

resul$tiempo <- system.time(resul$WinBUGS <- pbugs(data = datos, inits = iniciales,
    parameters.to.save = param, model = MmodelSA, n.iter = niters, n.burnin = nburns,
    n.chains = 3, DIC = F, pbugs.directory = path.expand("~/.wine/dosdevices/c:"),
    debug = F, bugs.seed = 1))


save(resul, file = "Data/Res_IsqWomen_9615_ALI_MmodelSA_30000it.Rdata")
```

Results: Convergence checking Mmodel SA Valencia

```
# Mean population Valencia 1996-2015 Women 9AgeGroups rounded zero
# decimals
load(file = "Data/MeanPop_Women_9AgeGroups_1996-2015_VAL.RData")
# pob[pob==0]<-1
pop.v <- pob
```

```r
rm(pob)
# Mortality Ischaemic Valencia 1996-2015 Women 9AgeGroup
load(file = "Data/Mortality_Ischaemic_Women_9AgeGroups_1996-2015_VAL.RData")
mort.v <- mort
rm(mort)
# In those census tracts, we change the population for the mortality
# pop.v[mort.v>pop.v] <- mort.v[mort.v>pop.v] Load results
load("Results/Res_IsqWomen_9615_VAL_modelSA_30000it.Rdata")
ResulModelv <- resul$WinBUGS
rm(resul)
# Load cartography
load("Data/Carto/carto_valencia.RData")

# TT are the number of years in the period of study.
TT <- 20

# convergence checking
p <- ResulModelv$summary[is.element(substr(row.names(ResulModelv$summary),
    1, 1), c("P")), ]
p1 <- p[order(p[, 8], decreasing = T), ][1:3, ]
p1 <- cbind(apply(p1[, 1:8], 2, round, digits = 3), p1[, 9])
colnames(p1)[9] <- "n.eff"
p2 <- p[order(p[, 9], decreasing = F), ][1:3, ]
p2 <- cbind(apply(p2[, 1:8], 2, round, digits = 3), p2[, 9])
colnames(p2)[9] <- "n.eff"
others.p <- as.data.frame.matrix(ResulModelv$summary[!is.element(substr(row.names(ResulModelv$summary),
    1, 1), c("P")), ])
others.p <- cbind(apply(others.p[, 1:8], 2, round, digits = 3), others.p[,
    9])
colnames(others.p)[9] <- "n.eff"
```

The Brooks-Gelman-Rubin statistic and the effective sample size are checked for all model parameters (4779 probabilities estimates, 531 census tracts x 9 age groups). Below are those estimates with higher values of Rhat or lower effective size.

	mean	sd	2.5%	25%	50%	75%	97.5%	Rhat	n.eff
P[520,7]	0.057	0.014	0.033	0.047	0.055	0.065	0.088	1.019	160
P[202,2]	0.002	0.001	0.001	0.001	0.002	0.002	0.003	1.018	120
P[492,3]	0.003	0.001	0.001	0.002	0.003	0.003	0.005	1.018	110

	mean	sd	2.5%	25%	50%	75%	97.5%	Rhat	n.eff
P[492,3]	0.003	0.001	0.001	0.002	0.003	0.003	0.005	1.018	110
P[202,2]	0.002	0.001	0.001	0.001	0.002	0.002	0.003	1.018	120
P[431,1]	0.002	0.000	0.001	0.001	0.002	0.002	0.003	1.015	130

For the rest of the parameters we get:

	mean	sd	2.5%	25%	50%	75%	97.5%	Rhat	n.eff
alpha[1]	-6.485	0.150	-6.769	-6.587	-6.485	-6.383	-6.199	1.002	1000
alpha[2]	-6.267	0.144	-6.561	-6.358	-6.264	-6.172	-5.992	1.002	860

	mean	sd	2.5%	25%	50%	75%	97.5%	Rhat	n.eff
alpha[3]	-5.918	0.133	-6.202	-6.002	-5.913	-5.822	-5.684	1.005	450
alpha[4]	-5.142	0.090	-5.321	-5.204	-5.136	-5.081	-4.971	1.001	1000
alpha[5]	-4.409	0.068	-4.550	-4.451	-4.409	-4.365	-4.276	1.000	1000
alpha[6]	-3.703	0.052	-3.815	-3.737	-3.703	-3.670	-3.603	1.002	860
alpha[7]	-2.880	0.042	-2.964	-2.906	-2.881	-2.852	-2.797	1.000	1000
alpha[8]	-2.118	0.037	-2.192	-2.141	-2.117	-2.093	-2.044	1.000	1000
alpha[9]	-1.066	0.033	-1.128	-1.087	-1.065	-1.046	-0.997	1.000	1000
gamma	0.717	0.204	0.183	0.639	0.771	0.860	0.947	1.009	250
ro2	0.579	0.133	0.299	0.500	0.586	0.675	0.809	1.009	260
sdstruct	0.596	0.077	0.450	0.544	0.599	0.646	0.748	1.018	180

Results: Convergence checking BYM model Valencia

```r
# Load results
load("Results/Res_IsqWomen_9615_VAL_ModeloBYMrepCarProper_30000it.Rdata")
ResulModelvBYMcp <- resul$WinBUGS
rm(resul)

smr <- ResulModelvBYMcp$summary[is.element(substr(row.names(ResulModelvBYMcp$summary),
    1, 3), c("RME")), ]
p1 <- smr[order(smr[, 8], decreasing = T), ][1:3, ]
p1 <- cbind(apply(p1[, 1:8], 2, round, digits = 3), p1[, 9])
colnames(p1)[9] <- "n.eff"
p2 <- smr[order(smr[, 9], decreasing = F), ][1:3, ]
p2 <- cbind(apply(p2[, 1:8], 2, round, digits = 3), p2[, 9])
colnames(p2)[9] <- "n.eff"
others.smr <- as.data.frame.matrix(ResulModelvBYMcp$summary[is.element(substr(row.names(ResulModelvBYMc
    1, 2), c("sd", "rh")), ])
others.smr <- cbind(apply(others.smr[, 1:8], 2, round, digits = 3), others.smr[,
    9])
colnames(others.smr)[9] <- "n.eff"
```

The Brooks-Gelman-Rubin statistic and the effective sample size are checked for all model parameters (531 smr estimates). Below are those estimates with higher values of Rhat or lower effective size.

	mean	sd	2.5%	25%	50%	75%	97.5%	Rhat	n.eff
RME[260]	100.685	11.450	79.546	93.385	100.15	107.200	125.000	1.012	210
RME[138]	97.059	9.675	79.160	90.615	97.32	103.200	117.597	1.011	210
RME[234]	105.608	12.919	81.772	97.175	104.55	113.275	133.992	1.010	220

	mean	sd	2.5%	25%	50%	75%	97.5%	Rhat	n.eff
RME[82]	105.673	12.508	83.445	97.020	104.60	112.900	133.897	1.009	200
RME[129]	102.175	10.081	83.946	95.450	101.90	108.375	122.697	1.009	210
RME[138]	97.059	9.675	79.160	90.615	97.32	103.200	117.597	1.011	210

For the rest of the parameters we get:

	mean	sd	2.5%	25%	50%	75%	97.5%	Rhat	n.eff
sdesp	0.259	0.050	0.161	0.227	0.258	0.292	0.358	1.005	1000
rho	0.833	0.276	-0.023	0.825	0.931	0.973	0.996	1.034	350

Results: Convergence checking Mmodel SA Alicante

```r
# Mean population Alicante 1996-2015 Women 9AgeGroups rounded zero
# decimals
load(file = "Data/MeanPop_Women_9AgeGroups_1996-2015_ALI.RData")
# pob[pob==0]<-1
pop.a <- pob
rm(pob)
# Mortality Ischaemic Alicante 1996-2015 Women 9AgeGroup
load(file = "Data/Mortality_Ischaemic_Women_9AgeGroups_1996-2015_ALI.RData")
mort.a <- mort
rm(mort)
# In those census tracts, we change the population for the mortality
pop.a[mort.a > pop.a] <- mort.a[mort.a > pop.a]
# Load results
load("Results/Res_IsqWomen_9615_ALI_modelSA_30000it.Rdata")
ResulModela <- resul$WinBUGS
rm(resul)
# Load cartography
load("Data/Carto/carto_alicante.RData")

# TT are the number of years in the period of study.
TT <- 20

# convergence checking
p <- ResulModela$summary[is.element(substr(row.names(ResulModela$summary),
    1, 1), c("P")), ]
p1 <- p[order(p[, 8], decreasing = T), ][1:3, ]
p1 <- cbind(apply(p1[, 1:8], 2, round, digits = 3), p1[, 9])
colnames(p1)[9] <- "n.eff"
p2 <- p[order(p[, 9], decreasing = F), ][1:3, ]
p2 <- cbind(apply(p2[, 1:8], 2, round, digits = 3), p2[, 9])
colnames(p2)[9] <- "n.eff"
others.p <- as.data.frame.matrix(ResulModela$summary[!is.element(substr(row.names(ResulModela$summary),
    1, 1), c("P")), ])
others.p <- cbind(apply(others.p[, 1:8], 2, round, digits = 3), others.p[,
    9])
colnames(others.p)[9] <- "n.eff"
```

The Brooks-Gelman-Rubin statistic and the effective sample size are checked for all model parameters (1602 probabilities estimates, 178 census tracts x 9 age groups). Below are those estimates with higher values of Rhat or lower effective size.

	mean	sd	2.5%	25%	50%	75%	97.5%	Rhat	n.eff
P[79,7]	0.057	0.015	0.032	0.046	0.056	0.067	0.092	1.017	110
P[21,4]	0.007	0.004	0.002	0.004	0.006	0.009	0.018	1.014	140
P[55,7]	0.054	0.022	0.022	0.038	0.050	0.065	0.108	1.012	190

	mean	sd	2.5%	25%	50%	75%	97.5%	Rhat	n.eff
P[79,7]	0.057	0.015	0.032	0.046	0.056	0.067	0.092	1.017	110
P[21,4]	0.007	0.004	0.002	0.004	0.006	0.009	0.018	1.014	140
P[69,7]	0.055	0.014	0.032	0.045	0.054	0.064	0.087	1.012	160

For the rest of the parameters we get:

	mean	sd	2.5%	25%	50%	75%	97.5%	Rhat	n.eff
alpha[1]	-6.591	0.258	-7.130	-6.755	-6.575	-6.425	-6.124	1.000	1000
alpha[2]	-6.037	0.203	-6.450	-6.170	-6.027	-5.902	-5.651	1.002	720
alpha[3]	-5.570	0.178	-5.919	-5.688	-5.566	-5.441	-5.246	1.000	1000
alpha[4]	-4.929	0.142	-5.221	-5.022	-4.931	-4.829	-4.654	1.000	1000
alpha[5]	-4.156	0.106	-4.377	-4.220	-4.153	-4.085	-3.944	1.003	620
alpha[6]	-3.511	0.088	-3.684	-3.569	-3.510	-3.456	-3.335	1.000	1000
alpha[7]	-2.698	0.077	-2.851	-2.747	-2.698	-2.649	-2.548	1.000	1000
alpha[8]	-1.749	0.067	-1.886	-1.793	-1.749	-1.706	-1.612	1.000	1000
alpha[9]	0.285	0.063	0.162	0.243	0.284	0.326	0.419	1.004	1000
gamma	0.433	0.278	-0.210	0.269	0.471	0.639	0.835	1.001	1000
ro2	0.474	0.101	0.260	0.405	0.478	0.545	0.664	1.003	710
sdstruct	1.146	0.088	0.966	1.090	1.148	1.205	1.309	1.004	1000

Results: Calculating sASRs

```
# Load standard population: European standard population 2013
pobest <- read.table("Data/European standard population 2013.txt", sep = " ",
    header = T)
# We select the age groups considered in this case study
pobestok <- pobest$StandPop[11:19]
grupoedad09 <- c("45-49", "50-54", "55-59", "60-64", "65-69", "70-74",
    "75-79", "80-84", ">=85")
names(pobestok) <- grupoedad09
W <- (pobestok/sum(pobestok)) * 1e+05


# ASR Valencia
# %%%%%%%%%%%%%%%%%%%%%%%%%%%%%%%%%%%%%%%%%%%%%%%%%%%%%%%%%%%%%%%%%%%%%%%%%%%%%%%%

resasr_VAL_Isq <- array(NA, dim = c(nrow(pop.v), 4, 2))
dimnames(resasr_VAL_Isq) <- list(SC = row.names(pop.v), Tasas = c("TB",
    "ASR", "ASRl", "ASRu"), RawSmoothed = c("Raw", "Smoothed"))

# raw age-specific mortality rates
rawR.v <- mort.v/(pop.v * TT)
# smoothed age-specific mortality rates
sPoDs.v <- apply(ResulModelv$sims.list$P, c(2, 3), mean)
smoR.v <- sPoDs.v/TT
row.names(smoR.v) <- row.names(mort.v)

for (i in row.names(pop.v)) {
    # raw ASR aux1<- sum(W*rawR.v[i,]) equivalent
```

```r
    aux1 <- ageadjust.direct(count = mort.v[i, ], pop = pop.v[i, ] * TT,
        stdpop = pobestok, conf.level = 0.95)
    resasr_VAL_Isq[match(i, dimnames(resasr_VAL_Isq)$SC), , "Raw"] <- round(as.numeric(aux1) *
        1e+05, 1)
    # aux2<- sum(W*smoR.v[i,]) equivalent
    aux2 <- ageadjust.direct(count = smoR.v[i, ] * (pop.v[i, ] * TT), pop = pop.v[i,
        ] * TT, stdpop = pobestok, conf.level = 0.95)
    resasr_VAL_Isq[match(i, dimnames(resasr_VAL_Isq)$SC), , "Smoothed"] <- round(as.numeric(aux2) *
        1e+05, 1)
}
# save(resasr_VAL_Isq,
# file='Results/Valencia9615_Isq_Women_resASR.RData')


# ASR Alicante
# %%%%%%%%%%%%%%%%%%%%%%%%%%%%%%%%%%%%%%%%%%%%%%%%%%%%%%%%%%%%%%%%%%%%%%%%%%%%%%

resasr_ALI_Isq <- array(NA, dim = c(nrow(pop.a), 4, 2))
dimnames(resasr_ALI_Isq) <- list(SC = row.names(pop.a), Tasas = c("TB",
    "ASR", "ASRl", "ASRu"), RawSmoothed = c("Raw", "Smoothed"))

# raw age-specific mortality rates
rawR.a <- mort.a/(pop.a * TT)
# smoothed age-specific mortality rates
sPoDs.a <- apply(ResulModela$sims.list$P, c(2, 3), mean)
smoR.a <- sPoDs.a/TT
row.names(smoR.a) <- row.names(mort.a)

for (i in row.names(pop.a)) {
    # raw ASR aux1<- sum(W*rawR.a[i,]) equivalent
    aux1 <- ageadjust.direct(count = mort.a[i, ], pop = pop.a[i, ] * TT,
        stdpop = pobestok, conf.level = 0.95)
    resasr_ALI_Isq[match(i, dimnames(resasr_ALI_Isq)$SC), , "Raw"] <- round(as.numeric(aux1) *
        1e+05, 1)
    # aux2<- sum(W*smoR.a[i,]) equiALIent
    aux2 <- ageadjust.direct(count = smoR.a[i, ] * (pop.a[i, ] * TT), pop = pop.a[i,
        ] * TT, stdpop = pobestok, conf.level = 0.95)
    resasr_ALI_Isq[match(i, dimnames(resasr_ALI_Isq)$SC), , "Smoothed"] <- round(as.numeric(aux2) *
        1e+05, 1)
}
# save(resasr_ALI_Isq,
# file='Results/Alicante9615_Isq_Women_resASR.RData')
```

Results: plot figure

```r
# postscript('../Articulo_Mmodelv4/FigurasASRisq/Mapas_Isq_Vlc_Densities_ASR_SMR_Quintiles_xaPaper2.eps
# width=9, height=9)

# Cartography
load("Data/Carto/carto_valencia.RData")
xlimok <- as.vector(summary(carto.valencia)$bbox["x", ]) - c(-0.02, 0.02)
ylimok <- as.vector(summary(carto.valencia)$bbox["y", ]) - c(-0.15, 0.08)
```

```r
par(mfrow = c(2, 2), mar = c(2, 2, 2, 2))

CortesFacFun <- function(dd, quintiles) {
    factor(as.numeric(Hmisc::cut2(dd, cuts = c(-Inf, quintiles, Inf))),
        levels = 1:5, labels = c(paste("[", format(ceiling(min(dd)), digits = 2,
            nsmall = 1), ", ", format(quintiles[1], digits = 2, nsmall = 1),
            ")", sep = ""), paste("[", format(quintiles[1], digits = 2,
            nsmall = 1), ", ", format(quintiles[2], digits = 2, nsmall = 1),
            ")", sep = ""), paste("[", format(quintiles[2], digits = 2,
            nsmall = 1), ", ", format(quintiles[3], digits = 2, nsmall = 1),
            ")", sep = ""), paste("[", format(quintiles[3], digits = 2,
            nsmall = 1), ", ", format(quintiles[4], digits = 2, nsmall = 1),
            ")", sep = ""), paste("[", format(quintiles[4], digits = 2,
            nsmall = 1), ", ", format(ceiling(max(dd)), digits = 2, nsmall = 1),
            ")", sep = "")))
}

# PLOT1: VLC ASMR
# %%%%%%%%%%%%%%%%%%%%%%%%%%%%%%%%%%%%%%%%%%%%%%%%%%%%%%%%%%%%%%%%%%%%%%%%%%%%%%%
datos <- resasr_VAL_Isq[, "ASR", "Raw"]
quintiles <- as.numeric(quantile(x = resasr_VAL_Isq[, "ASR", "Smoothed"],
    probs = seq(0, 1, length.out = 6)[-c(1, 6)], type = 4))
CortesFac <- CortesFacFun(datos, quintiles)
Cortes <- CortesFac
valores <- as.numeric(CortesFac)
mypalette <- RColorBrewer::brewer.pal(length(levels(CortesFac)), "Blues")
fgs <- mypalette[valores]

plot(carto.valencia, col = fgs, xlab = "", ylab = "", axes = F, xlim = xlimok,
    ylim = ylimok)
auxgrafcompesp <- matrix(c(levels(CortesFac), paste("(", as.numeric(table(factor(valores,
    levels = 1:5, labels = 1:5))), ")", sep = "")), nrow = 5, ncol = 2)
legend("topright", legend = apply(auxgrafcompesp, 1, paste, collapse = " "),
    fill = mypalette, border = mypalette, cex = 0.6, title = paste0("ASR x",
        expression(10^5)), bty = "n")
title("Raw ASR Valencia", cex.main = 1)


# PLOT2: VLC sASMR
# %%%%%%%%%%%%%%%%%%%%%%%%%%%%%%%%%%%%%%%%%%%%%%%%%%%%%%%%%%%%%%%%%%%%%%%%%%%%%%%
datos <- resasr_VAL_Isq[, "ASR", "Smoothed"]
quintiles <- as.numeric(quantile(x = resasr_VAL_Isq[, "ASR", "Smoothed"],
    probs = seq(0, 1, length.out = 6)[-c(1, 6)], type = 4))
CortesFac <- CortesFacFun(datos, quintiles)
Cortes <- CortesFac
valores <- as.numeric(CortesFac)
mypalette <- RColorBrewer::brewer.pal(length(levels(CortesFac)), "Blues")
fgs <- mypalette[valores]

plot(carto.valencia, col = fgs, xlab = "", ylab = "", axes = F, xlim = xlimok,
    ylim = ylimok)
auxgrafcompesp <- matrix(c(levels(CortesFac), paste("(", as.numeric(table(factor(valores,
    levels = 1:5, labels = 1:5))), ")", sep = "")), nrow = 5, ncol = 2)
```

```r
legend("topright", legend = apply(auxgrafcompesp, 1, paste, collapse = " "),
    fill = mypalette, border = mypalette, cex = 0.6, title = paste0("sASR x",
        expression(10^5)), bty = "n")
title("Smoothed ASR Valencia", cex.main = 1)


# PLOT3: VLC SMR CP
# %%%%%%%%%%%%%%%%%%%%%%%%%%%%%%%%%%%%%%%%%%%%%%%%%%%%%%%%%%%%%%%%%%%%%%%%%%%%%%%%
datos <- ResulModelvBYMcp$mean$RME
quintiles <- as.numeric(quantile(x = datos, seq(0, 1, length.out = 6)[-c(1,
    6)], type = 4))
CortesFac <- CortesFacFun(datos, quintiles)
Cortes <- CortesFac
valores <- as.numeric(CortesFac)
mypalette <- RColorBrewer::brewer.pal(length(levels(CortesFac)), "Blues")
fgs <- mypalette[valores]

plot(carto.valencia, col = fgs, xlab = "", ylab = "", axes = F, xlim = xlimok,
    ylim = ylimok)
auxgrafcompesp <- matrix(c(levels(CortesFac), paste("(", as.numeric(table(factor(valores,
    levels = 1:5, labels = 1:5))), ")", sep = "")), nrow = 5, ncol = 2)
legend("topright", legend = apply(auxgrafcompesp, 1, paste, collapse = " "),
    fill = mypalette, border = mypalette, cex = 0.6, title = paste0("SMR"),
    bty = "n")
title("Smoothed SMR Valencia", cex.main = 1)



# PLOT4: Densities
# %%%%%%%%%%%%%%%%%%%%%%%%%%%%%%%%%%%%%%%%%%%%%%%%%%%%%%%%%%%%%%%%%%%%%%%%%%%%%%%%
par(mar = c(5, 5, 3, 4))
plot(density(resasr_ALI_Isq[, "ASR", "Smoothed"]), col = 1, lty = 2, lwd = 2,
    xlab = "sASR", xlim = c(100, 350), ylim = c(0, 0.05), bty = "l", main = "")
lines(density(resasr_VAL_Isq[, "ASR", "Smoothed"]), col = 1, lty = 1, lwd = 2)
legend(200, 0.05, legend = c("Valencia", "Alicante"), lty = c(1, 2), lwd = 2,
    bty = "n")
```

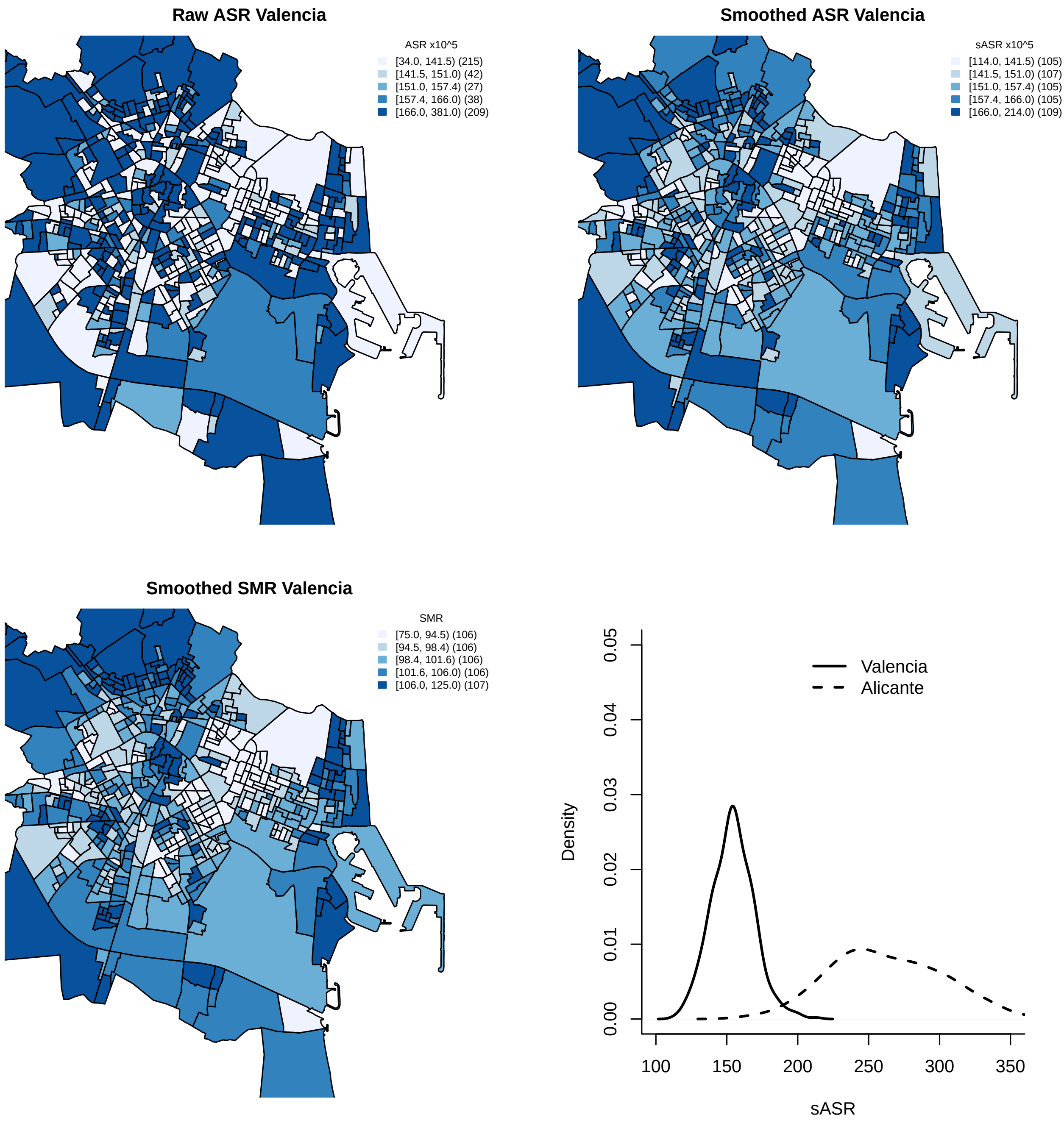


```r
# dev.off()
```

Fig 4. Smoothed age standardized mortality rate from ischaemic heart disease in Valencia and Alicante. Women over 45 years, period 1996-2015.

Results: comparing the risks of two particular census tracts

```r
# Extreme values
mintract <- which((resasr_VAL_Isq[, "ASR", "Smoothed"] == min(resasr_VAL_Isq[,
    "ASR", "Smoothed"])))
```

```r
maxtract <- which((resasr_VAL_Isq[, "ASR", "Smoothed"] == max(resasr_VAL_Isq[,
    "ASR", "Smoothed"])))

minsim <- ResulModelv$sims.list$P[, mintract, ]
maxsim <- ResulModelv$sims.list$P[, maxtract, ]

asr_minmax <- data.frame(iter = 1:1002, sASRmin = NA, sASRmax = NA)

# Rellenamos el dataframe
for (i in 1:1002) {
    aux2 <- ageadjust.direct(pop = pop.v[mintract, ], count = (minsim[i,
        ]/20) * pop.v[mintract, ], stdpop = pobestok, conf.level = 0.95)
    asr_minmax[i, "sASRmin"] <- round(as.numeric(aux2[2]) * 1e+05, 1)
    aux3 <- ageadjust.direct(pop = pop.v[maxtract, ], count = (maxsim[i,
        ]/20) * pop.v[maxtract, ], stdpop = pobestok, conf.level = 0.95)
    asr_minmax[i, "sASRmax"] <- round(as.numeric(aux3[2]) * 1e+05, 1)
}

# plot(density(asr_minmax[,'sASRmin']), col=1, lty=2,lwd=2,
# xlab='sASMR', xlim=c(100,700), ylim=c(0,0.02), bty='l', main='')
# lines(density(asr_minmax[,'sASRmax']), col=1, lty=1, lwd=2)

sum(asr_minmax[, "sASRmax"] > asr_minmax[, "sASRmin"])/1002
```

```
## [1] 0.996008
```

Results: correlation ASR-SMR and quantiles

```r
cor(resasr_VAL_Isq[, "ASR", "Smoothed"], ResulModelvBYMcp$mean$RME, method = c("pearson"))
```

```
## [1] 0.9456984
```

As shown in that figure, most of the Valencia census tracts have sASR values smaller than any of the Alicante census tracts. As a matter of percentiles, the 95% percentile of the sASR distribution in Valencia (178.9 deaths per 100,000 women) is lower than the 5% percentile for Alicante (203.5).

Quantile Valencia

0%	5%	10%	15%	20%	25%	30%	35%	40%	45%	50%
113.2	129.9	135.4	138.7	141.5	143.9	146.5	148.8	151.1	152.4	154

55%	60%	65%	70%	75%	80%	85%	90%	95%	100%
155.5	157.4	159	161.4	164.1	166	169	172.1	178.9	213.1

Quantile Alicante

0%	5%	10%	15%	20%	25%	30%	35%	40%	45%	50%
169	203.5	216.7	223.7	227.7	234.7	240	244.4	249.3	254.1	258.4

55%	60%	65%	70%	75%	80%	85%	90%	95%	100%
267	272.8	279.1	284.4	291.6	301.3	308.2	313.4	331.3	422.8

Case Study 3: smoothed life expectancies.

Life expectancy estimation

```
# FUNCTION: Life expectancy estimation 19 age groups
# %%%%%%%%%%%%%%%%%%%%%%%%%%%%%%%%%%%%%%%%%%%%%%%%%%%%%%%%%%%%%%%%%%%%%%%%%%%%%%%%

# PoDs = estimated probabilities from Mmodel TT = number of years in
# the period of study.

LEfun <- function(PoDs, TT) {
    # Age interval
    agegroups <- c("0", "1-4", "5-9", "10-14", "15-19", "20-24", "25-29",
        "30-34", "35-39", "40-44", "45-49", "50-54", "55-59", "60-64",
        "65-69", "70-74", "75-79", "80-84", ">=85")
    # Probs
    P <- as.numeric(PoDs)
    # Age at start of interval
    x <- c(0, 1, 5, 10, 15, 20, 25, 30, 35, 40, 45, 50, 55, 60, 65, 70,
        75, 80, 85)
    # Interval width
    n <- c(1, 4, 5, 5, 5, 5, 5, 5, 5, 5, 5, 5, 5, 5, 5, 5, 5, 5, NA)
    # Oldest age group width is taken to be twice the mean survival in this
    # age group assuming exponential survival, $n_A=2/R_{sA}$.
    n[19] <- 2 * TT/P[19]
    # Fraction of last age interval
    a <- c(0.1, rep(0.5, 18))
    # Mean age of death
    age <- x + n * a

    LEcomp <- rep(NA, 19)
    LEcomp[1] <- age[1] * P[1]
    for (j in 2:18) {
        LEcomp[j] <- age[j] * P[j] * prod((1 - P)[1:(j - 1)])
    }
    LEcomp[19] <- age[19] * prod((1 - P)[1:18])

    return(sum(LEcomp))

}
```

```r
# FUNCTION: Life expectancy estimation from Life table Chiang II
# adjusted
# %%%%%%%%%%%%%%%%%%%%%%%%%%%%%%%%%%%%%%%%%%%%%%%%%%%%%%%%%%%%%%%%%%%%%%%%%%%%%%%%

LifeTableChiangIIadj <- function(Pob, Mort) {
    agegroups <- c("0", "1-4", "5-9", "10-14", "15-19", "20-24", "25-29",
        "30-34", "35-39", "40-44", "45-49", "50-54", "55-59", "60-64",
        "65-69", "70-74", "75-79", "80-84", ">=85")

    # Interval Width
    n <- c(1, 4, 5, 5, 5, 5, 5, 5, 5, 5, 5, 5, 5, 5, 5, 5, 5, 5, NA)

    ngredad <- length(agegroups)
    TV <- data.frame(matrix(NA, nrow = ngredad, ncol = 12))
    colnames(TV) <- c("AgeGroup", "n", "a", "PopYears", "Deaths", "M",
        "q", "l", "d", "L", "T", "EV")

    # Age Group labels
    TV$AgeGroup <- agegroups

    # a = Fraction of Last Age Interval
    TV$a <- c(0.1, rep(0.5, (ngredad - 1)))

    # Population Years at Risk
    TV$PopYears <- as.numeric(Pob)

    # Number of Deaths in Interval
    TV$Deaths <- as.numeric(Mort)

    # Death Rate in Interval
    TV$M <- TV$Deaths/TV$PopYears

    # The final age interval is open ended. However since L_w = 1/M_w and
    # can also be written as n_i*a_i, setting a to 0.5 (as for the other
    # age intervals gives a hypothetical width n_w = 2 / M_w
    TV$n <- n
    TV$n[ngredad] <- 1/TV$a[ngredad]/TV$M[ngredad]

    # Probability of Dying in Interval
    TV$q <- (TV$n * TV$M)/(1 + (1 - TV$a) * TV$n * TV$M)
    TV$q[TV$Deaths > TV$PopYears/TV$n/TV$a] <- 1
    TV$q[ngredad] <- 1

    # Number Alive at Start of Interval
    TV$l[1] <- 1e+05
    for (i in 1:(ngredad - 1)) {
        # d Number Dying in Interval
        TV$d[i] <- TV$q[i] * TV$l[i]
        TV$l[i + 1] <- TV$l[i] - TV$d[i]
    }
    TV$d[ngredad] <- TV$q[ngredad] * TV$l[ngredad]
```

```
    # Person-Years Lived in Interval
    TV$L <- (TV$n * (TV$l - TV$d)) + (TV$a * TV$n * TV$d)
    TV$L[ngredad] <- TV$l[ngredad]/TV$M[ngredad]

    # Person-Years Lived Beyond Start of Interval
    TV$T <- c(sum(TV$L), sum(TV$L) - cumsum(TV$L))[-(length(TV$L) + 1)]

    # Observed Life Expectancy at Start of Interval
    TV$EV <- TV$T/TV$l

    # # Sample Variance of Proportion Surviving in Interval TV$Sp <-
    # ifelse( TV$Deaths==0, 0, TV$q^2*(1-TV$q)/TV$Deaths ) TV$Sp[ngredad]
    # <- 4 / TV$Deaths[ngredad] / TV$M[ngredad]^2 # Weighted Variance of
    # Proportion Surviving in Interval for (i in 1:(ngredad-1)) {TV$WSp[i]
    # <- TV$l[i]^2 * ( (1-TV$a[i]) * TV$n[i] + TV$EV[i+1])^2 * TV$Sp[i]}
    # TV$WSp[ngredad] <- (TV$l[ngredad]/2)^2 * TV$Sp[ngredad] # Sample
    # Variance of Person-Years Lived Beyond Start of Interval
    # TV$ST[ngredad] <- TV$WSp[ngredad] for (i in (ngredad-1):1) {TV$ST[i]
    # <- TV$ST[i+1] + TV$WSp[i]} # Sample Variance of Observed Life
    # Expectancy at Start of Interval TV$se2 <- TV$ST / TV$l^2 # 95%
    # Confidence Interval for Observed Life Expectancy at Start of Interval
    # TV$ICEVlo <- TV$EV-1.96*sqrt(TV$se2) TV$ICEVup <-
    # TV$EV+1.96*sqrt(TV$se2)

    return(TV)
}
# 'PHE Life Expectancy Calculator.xlsm' Chiang(II) adjusted Silcocks
```

Running the models

```
# Data Valencia men
# %%%%%%%%%%%%%%%%%%%%%%%%%%%%%%%%%%%%%%%%%%%%%%%%%%%%%%%%%%%%%%%%%%%%%%%%%%%%%%

# Global men mortality Comunitat Valenciana (Spain).  Period 2014-2017
# Spatial units of study: 542 municipalities (Comunitat Valenciana,
# Spain) Age: 19 age intervals.

# Mean population Comunitat Valenciana 2014-2017 rounded zero decimals
load(file = "Data/MeanPob_Men_19AgeGroups_2014-2017_CV.RData")
# Global men mortality Comunitat Valenciana 2014-2017 19AgeGroup
load(file = "Data/Mortality_Global_Men_19AgeGroups_2014-2017_CV.RData")

dim(mort)
dim(pob)
# [1] 542 19 [1] 542 19

# There are a total of 88,695 deaths for the whole period of study.
sum(mort)
# 88695

# Age groups of municipalities whose population is zero in the study
# period are replaced by 1 for correct modelling with binomial
```

```r
# distribution.  There are 272 AgeGroup-Areas with zero population in
# 10298 combinations.
length(pob[pob == 0])
# [1] 272
pob[pob == 0] <- 1

# We observe 24 combinations AgeGroup-Areas in which the mortality is
# higher than its mean population.
mort[mort > pob]
# [1] 7 2 11 3 2 30 23 2 5 4 4 4 24 2 6 10 16 4 15 14 2 42 8 9
pob[mort > pob]
# [1] 3 1 9 1 1 29 20 1 2 3 3 3 23 1 4 9 14 2 14 12 1 41 6 3

# In those combinations AgeGroup-Areas, we change the population for
# the mortality
pob[mort > pob] <- mort[mort > pob]

pob <- data.matrix(pob)
mort <- data.matrix(mort)



# Preparing and running the model
# %%%%%%%%%%%%%%%%%%%%%%%%%%%%%%%%%%%%%%%%%%%%%%%%%%%%%%%%%%%%%%%%%%%%%%%%%%%%%%

nGroups <- dim(mort)[1:2]  #SC,GEdad = 542 19
NSC <- dim(mort)[1]
NGed <- dim(mort)[2]

# Cartography
carto <- rgdal::readOGR(dsn = "Data/Carto", layer = "mapa_cv_muni", verbose = FALSE)
sp::proj4string(carto) <- sp::CRS("+proj=longlat +datum=WGS84")
index <- order(carto$INE_MUN)
carto <- carto[index, ]

# Neighborhood structure is created for the CAR.normal distribution.
Veci <- spdep::poly2nb(carto)

# Neighbourhood relations are added to join an 'island' of the
# cartography of the Valencian Community to the municipalities closest
# to it.
Veci[[317]] <- as.integer(c(Veci[[317]], 364))
Veci[[364]] <- as.integer(c(Veci[[364]], 317))
Veci[[477]] <- as.integer(c(Veci[[477]], 312))
Veci[[312]] <- as.integer(c(Veci[[312]], 477))

# Convert neighborhood structure to Winbugs
VeciWB <- spdep::nb2WB(Veci)

datos <- list(Obs = mort[, ], N = pob[, ], nGroups = nGroups, adj = VeciWB$adj,
    num = VeciWB$num, C = rep(1/VeciWB$num, VeciWB$num), M = 1/VeciWB$num)

set.seed(1)
```

```
# alpha is initialized into the mean values by age group to accelerate
# model convergence.
medias <- matrix(as.numeric(round(logit(apply(mort/pob, 2, mean)), 0)),
    nrow = nGroups[2], ncol = 1, byrow = F)
iniciales <- function() {
    list(alpha = rnorm(nGroups[2], medias, 0.5), gamma = runif(1, 0.4,
        0.6), ro2 = runif(1, 0.5, 1), sdstruct = runif(1, 0.2, 0.5), tS1 = array(rnorm(nGroups[1] *
        nGroups[2], 0, 1), dim = c(nGroups[2], nGroups[1])))
}


param <- c("P", "alpha", "gamma", "ro2", "sdstruct")

niters <- 30000
nburns <- 5000

resul <- list()

resul$tiempo <- system.time(resul$WinBUGS <- pbugs(data = datos, inits = iniciales,
    parameters.to.save = param, model = modelo_seleccionado, n.iter = niters,
    n.burnin = nburns, n.chains = 3, DIC = F, pbugs.directory = path.expand("~/.wine/dosdevices/c:"),
    debug = F, bugs.seed = 1))


save(resul, file = "Data/Res_GlobalMen_1417_CV_MmodelSA_30000it.Rdata")



# Data Valencia women
# %%%%%%%%%%%%%%%%%%%%%%%%%%%%%%%%%%%%%%%%%%%%%%%%%%%%%%%%%%%%%%%%%%%%%%%%%%%%%%%%

# Global women mortality Comunitat Valenciana (Spain).  Period
# 2014-2017 Spatial units of study: 542 municipalities (Comunitat
# Valenciana, Spain) Age: 19 age intervals.

# Mean population Comunitat Valenciana 2014-2017 rounded zero decimals
load(file = "Data/MeanPob_Women_19AgeGroups_2014-2017_CV.RData")
# Global women mortality Comunitat Valenciana 2014-2017 19AgeGroup
load(file = "Data/Mortality_Global_Women_19AgeGroups_2014-2017_CV.RData")

dim(mort)
dim(pob)
# [1] 542 19 [1] 542 19

# There are a total of 84669 deaths for the whole period of study.
sum(mort)
# 84669

# Age groups of municipalities whose population is zero in the study
# period are replaced by 1 for correct modelling with binomial
# distribution.  There are 308 AgeGroup-Areas with zero population in
# 10298 combinations.
length(pob[pob == 0])
```

```r
# [1] 308
pob[pob == 0] <- 1

# We observe 8 combinations AgeGroup-Areas in which the mortality is
# higher than its mean population.
mort[mort > pob]
# [1] 4 2 6 13 5 19 3 14
pob[mort > pob]
# [1] 3 1 3 10 4 17 2 7

# In those combinations AgeGroup-Areas, we change the population for
# the mortality
pob[mort > pob] <- mort[mort > pob]

pob <- data.matrix(pob)
mort <- data.matrix(mort)



# Preparing and running the model
# %%%%%%%%%%%%%%%%%%%%%%%%%%%%%%%%%%%%%%%%%%%%%%%%%%%%%%%%%%%%%%%%%%%%%%%%%%%%%%

nGroups <- dim(mort)[1:2]  #SC,GEdad = 542 19
NSC <- dim(mort)[1]
NGed <- dim(mort)[2]

# Cartography
carto <- rgdal::readOGR(dsn = "Data/Carto", layer = "mapa_cv_muni", verbose = FALSE)
sp::proj4string(carto) <- sp::CRS("+proj=longlat +datum=WGS84")
index <- order(carto$INE_MUN)
carto <- carto[index, ]

# Neighborhood structure is created for the CAR.normal distribution.
Veci <- spdep::poly2nb(carto)

# Neighbourhood relations are added to join an 'island' of the
# cartography of the Valencian Community to the municipalities closest
# to it.
Veci[[317]] <- as.integer(c(Veci[[317]], 364))
Veci[[364]] <- as.integer(c(Veci[[364]], 317))
Veci[[477]] <- as.integer(c(Veci[[477]], 312))
Veci[[312]] <- as.integer(c(Veci[[312]], 477))

# Convert neighborhood structure to Winbugs
VeciWB <- spdep::nb2WB(Veci)

datos <- list(Obs = mort[, ], N = pob[, ], nGroups = nGroups, adj = VeciWB$adj,
    num = VeciWB$num, C = rep(1/VeciWB$num, VeciWB$num), M = 1/VeciWB$num)

set.seed(1)

# alpha is initialized into the mean values by age group to accelerate
# model convergence.
```

```r
medias <- matrix(as.numeric(round(logit(apply(mort/pob, 2, mean)), 0)),
    nrow = nGroups[2], ncol = 1, byrow = F)
iniciales <- function() {
    list(alpha = rnorm(nGroups[2], medias, 0.5), gamma = runif(1, 0.4,
        0.6), ro2 = runif(1, 0.5, 1), sdstruct = runif(1, 0.2, 0.5), tS1 = array(rnorm(nGroups[1] *
        nGroups[2], 0, 1), dim = c(nGroups[2], nGroups[1])))
}


param <- c("P", "alpha", "gamma", "ro2", "sdstruct")

niters <- 30000
nburns <- 5000

resul <- list()

resul$tiempo <- system.time(resul$WinBUGS <- pbugs(data = datos, inits = iniciales,
    parameters.to.save = param, model = modelo_seleccionado, n.iter = niters,
    n.burnin = nburns, n.chains = 3, DIC = F, pbugs.directory = path.expand("~/.wine/dosdevices/c:"),
    debug = F, bugs.seed = 1))


save(resul, file = "Data/Res_GlobalWomen_1417_CV_MmodelSA_30000it.Rdata")
```

Results: Convergence checking Mmodel SA Men

```r
# TT = number of years in the period of study.
TT <- 4

# Mean population CV 2014-2017 Men 19AgeGroups rounded zero decimals
load(file = "Data/MeanPob_Men_19AgeGroups_2014-2017_CV.RData")
popM <- pob
rm(pob)
# Person Years Men
popYM <- popM * TT
popYM[popYM == 0] <- 1

# Global mortality CV 2014-2017 Men 19AgeGroups
load(file = "Data/Mortality_Global_Men_19AgeGroups_2014-2017_CV.RData")
mortM <- mort
rm(mort)
# In those census tracts, we change the population for the mortality
popM[mortM > popM] <- mortM[mortM > popM]
# Load results
load("Results/Res_GlobalMen_1417_CV_modelSA_30000it.Rdata")
ResulModelM <- resul$WinBUGS
rm(resul)
# Load cartography
carto <- rgdal::readOGR(dsn = "Data/Carto", layer = "mapa_cv_muni", verbose = FALSE)
sp::proj4string(carto) <- sp::CRS("+proj=longlat +datum=WGS84")
index <- order(carto$INE_MUN)
carto <- carto[index, ]
```

```r
# convergence checking
p <- ResulModelM$summary[is.element(substr(row.names(ResulModelM$summary),
    1, 1), c("P")), ]
p1 <- p[order(p[, 8], decreasing = T), ][1:3, ]
p1 <- cbind(apply(p1[, 1:8], 2, round, digits = 3), p1[, 9])
colnames(p1)[9] <- "n.eff"
p2 <- p[order(p[, 9], decreasing = F), ][1:3, ]
p2 <- cbind(apply(p2[, 1:8], 2, round, digits = 3), p2[, 9])
colnames(p2)[9] <- "n.eff"
others.p <- as.data.frame.matrix(ResulModelM$summary[!is.element(substr(row.names(ResulModelM$summary),
    1, 1), c("P")), ])
others.p <- cbind(apply(others.p[, 1:8], 2, round, digits = 3), others.p[,
    9])
colnames(others.p)[9] <- "n.eff"
```

The Brooks-Gelman-Rubin statistic and the effective sample size are checked for all model parameters (10298 probabilities estimates, 542 municipalities x 19 age groups). Below are those estimates with higher values of Rhat or lower effective size.

	mean	sd	2.5%	25%	50%	75%	97.5%	Rhat	n.eff
P[65,17]	0.161	0.006	0.150	0.157	0.161	0.165	0.174	1.017	130
P[540,18]	0.286	0.040	0.212	0.258	0.285	0.314	0.370	1.017	210
P[477,5]	0.001	0.000	0.000	0.001	0.001	0.001	0.002	1.016	140

	mean	sd	2.5%	25%	50%	75%	97.5%	Rhat	n.eff
P[65,17]	0.161	0.006	0.150	0.157	0.161	0.165	0.174	1.017	130
P[263,11]	0.009	0.002	0.006	0.007	0.009	0.010	0.013	1.015	130
P[56,17]	0.168	0.013	0.146	0.159	0.168	0.177	0.195	1.014	140

For the rest of the parameters we get:

	mean	sd	2.5%	25%	50%	75%	97.5%	Rhat	n.eff
alpha[1]	-4.350	0.072	-4.483	-4.400	-4.348	-4.302	-4.210	1.002	920
alpha[2]	-7.689	0.153	-8.014	-7.793	-7.685	-7.587	-7.399	1.001	1000
alpha[3]	-8.005	0.153	-8.324	-8.106	-8.003	-7.907	-7.716	1.002	740
alpha[4]	-8.206	0.178	-8.570	-8.321	-8.197	-8.071	-7.895	1.001	1000
alpha[5]	-6.993	0.103	-7.201	-7.063	-6.987	-6.925	-6.793	1.007	510
alpha[6]	-6.714	0.089	-6.887	-6.773	-6.717	-6.657	-6.543	1.002	830
alpha[7]	-6.682	0.084	-6.852	-6.736	-6.679	-6.625	-6.525	1.002	960
alpha[8]	-6.278	0.067	-6.407	-6.323	-6.278	-6.236	-6.157	1.002	830
alpha[9]	-5.946	0.056	-6.058	-5.982	-5.945	-5.910	-5.841	1.005	410
alpha[10]	-5.318	0.046	-5.409	-5.348	-5.317	-5.285	-5.229	1.012	210
alpha[11]	-4.741	0.041	-4.820	-4.769	-4.744	-4.714	-4.657	1.007	310
alpha[12]	-4.117	0.036	-4.184	-4.143	-4.118	-4.093	-4.047	1.009	220
alpha[13]	-3.609	0.033	-3.673	-3.631	-3.610	-3.587	-3.545	1.006	290
alpha[14]	-3.193	0.033	-3.254	-3.216	-3.193	-3.171	-3.122	1.006	290
alpha[15]	-2.765	0.032	-2.828	-2.787	-2.765	-2.744	-2.701	1.006	310
alpha[16]	-2.299	0.032	-2.361	-2.322	-2.300	-2.278	-2.234	1.002	720
alpha[17]	-1.696	0.032	-1.759	-1.716	-1.694	-1.675	-1.631	1.003	560

	mean	sd	2.5%	25%	50%	75%	97.5%	Rhat	n.eff
alpha[18]	-0.951	0.031	-1.011	-0.971	-0.950	-0.931	-0.891	1.006	320
alpha[19]	0.635	0.032	0.573	0.614	0.634	0.657	0.697	1.006	380
gamma	0.882	0.041	0.795	0.858	0.885	0.912	0.950	1.003	560
ro2	0.836	0.023	0.790	0.821	0.836	0.852	0.879	1.001	1000
sdstruct	0.484	0.026	0.434	0.465	0.483	0.500	0.538	1.009	230

Results: Convergence checking Mmodel SA Women

```
# Mean population CV 2014-2017 Women 19AgeGroups rounded zero decimals
load(file = "Data/MeanPob_Women_19AgeGroups_2014-2017_CV.RData")
popW <- pob
rm(pob)
# Person Years Men
popYW <- popW * TT
popYW[popYW == 0] <- 1
# Global mortality CV 2014-2017 Women 19AgeGroups
load(file = "Data/Mortality_Global_Women_19AgeGroups_2014-2017_CV.RData")
mortW <- mort
rm(mort)
# In those census tracts with mort>pop, we change the population for
# the mortality
popW[mortW > popW] <- mortW[mortW > popW]
# Load results
load("Results/Res_GlobalWomen_1417_CV_modelSA_30000it.Rdata")
ResulModelW <- resul$WinBUGS
rm(resul)

# convergence checking
p <- ResulModelW$summary[is.element(substr(row.names(ResulModelW$summary),
    1, 1), c("P")), ]
p1 <- p[order(p[, 8], decreasing = T), ][1:3, ]
p1 <- cbind(apply(p1[, 1:8], 2, round, digits = 3), p1[, 9])
colnames(p1)[9] <- "n.eff"
p2 <- p[order(p[, 9], decreasing = F), ][1:3, ]
p2 <- cbind(apply(p2[, 1:8], 2, round, digits = 3), p2[, 9])
colnames(p2)[9] <- "n.eff"
others.p <- as.data.frame.matrix(ResulModelW$summary[!is.element(substr(row.names(ResulModelW$summary),
    1, 1), c("P")), ])
others.p <- cbind(apply(others.p[, 1:8], 2, round, digits = 3), others.p[,
    9])
colnames(others.p)[9] <- "n.eff"
```

The Brooks-Gelman-Rubin statistic and the effective sample size are checked for all model parameters (10298 probabilities estimates, 542 municipalities x 19 age groups). Below are those estimates with higher values of Rhat or lower effective size.

	mean	sd	2.5%	25%	50%	75%	97.5%	Rhat	n.eff
P[486,17]	0.082	0.019	0.051	0.068	0.080	0.093	0.125	1.019	120
P[503,19]	0.555	0.050	0.455	0.520	0.556	0.590	0.651	1.018	180
P[201,10]	0.003	0.001	0.001	0.002	0.003	0.003	0.005	1.016	120

	mean	sd	2.5%	25%	50%	75%	97.5%	Rhat	n.eff
P[201,9]	0.002	0.001	0.001	0.001	0.002	0.002	0.003	1.016	120
P[201,10]	0.003	0.001	0.001	0.002	0.003	0.003	0.005	1.016	120
P[486,17]	0.082	0.019	0.051	0.068	0.080	0.093	0.125	1.019	120

For the rest of the parameters we get:

	mean	sd	2.5%	25%	50%	75%	97.5%	Rhat	n.eff
alpha[1]	-4.604	0.071	-4.744	-4.652	-4.605	-4.555	-4.473	1.001	1000
alpha[2]	-7.521	0.141	-7.804	-7.616	-7.513	-7.424	-7.254	1.000	1000
alpha[3]	-8.446	0.191	-8.837	-8.577	-8.439	-8.311	-8.104	1.005	380
alpha[4]	-8.290	0.178	-8.657	-8.403	-8.286	-8.168	-7.960	1.005	510
alpha[5]	-7.697	0.147	-8.005	-7.794	-7.690	-7.593	-7.422	1.000	1000
alpha[6]	-7.674	0.130	-7.925	-7.759	-7.669	-7.582	-7.436	1.002	960
alpha[7]	-7.438	0.115	-7.667	-7.516	-7.442	-7.358	-7.225	1.001	1000
alpha[8]	-7.067	0.084	-7.242	-7.123	-7.064	-7.007	-6.912	1.001	1000
alpha[9]	-6.461	0.057	-6.572	-6.497	-6.460	-6.425	-6.344	1.006	460
alpha[10]	-5.867	0.046	-5.962	-5.900	-5.866	-5.836	-5.781	1.003	1000
alpha[11]	-5.347	0.038	-5.419	-5.372	-5.347	-5.321	-5.272	1.012	170
alpha[12]	-4.826	0.032	-4.889	-4.847	-4.825	-4.805	-4.764	1.003	740
alpha[13]	-4.403	0.030	-4.465	-4.423	-4.402	-4.382	-4.347	1.004	460
alpha[14]	-4.093	0.027	-4.145	-4.110	-4.095	-4.075	-4.035	1.002	840
alpha[15]	-3.681	0.024	-3.728	-3.698	-3.680	-3.664	-3.635	1.000	1000
alpha[16]	-3.120	0.021	-3.164	-3.134	-3.119	-3.106	-3.081	1.003	620
alpha[17]	-2.381	0.018	-2.417	-2.393	-2.380	-2.368	-2.345	1.003	560
alpha[18]	-1.516	0.017	-1.548	-1.527	-1.516	-1.506	-1.483	1.002	1000
alpha[19]	0.254	0.015	0.225	0.243	0.254	0.264	0.286	1.001	1000
gamma	0.267	0.244	-0.232	0.091	0.274	0.466	0.681	1.003	630
ro2	0.734	0.048	0.627	0.706	0.737	0.766	0.817	1.003	1000
sdstruct	0.453	0.026	0.400	0.436	0.453	0.470	0.502	1.003	500

Results: LE estimates

```
LE <- array(NA, dim = c(542, 2, 2))
dimnames(LE) <- list(row.names(popM), c("Raw", "Smoothed"), c("Men", "Women"))

# Raw LE estimates are calculated by life table ChiangII adjusted using
# original data.  Population CV 2014-2017 Men 19AgeGroups rounded zero
# decimals
load(file = "Data/Pob_Men_19AgeGroups_2014-2017_CV.RData")
popM1417[popM1417 == 0] <- 1
popM1417[popM1417 < mortM]
```

```
## [1] 1
```

```
mortM[popM1417 < mortM]
```

```
## [1] 2
```

```
popM1417[popM1417 < mortM] <- mortM[popM1417 < mortM]
```

```r
# Population CV 2014-2017 Women 19AgeGroups rounded zero decimals
load(file = "Data/Pob_Women_19AgeGroups_2014-2017_CV.RData")
popW1417[popW1417 == 0] <- 1
# popW1417[popW1417<mortW] numeric(0)

for (i in row.names(popM)) {
    LE[dimnames(LE)[[1]] == i, "Raw", "Men"] <- LifeTableChiangIIadj(popM1417[i,
        ], mortM[i, ])$EV[1]
    LE[dimnames(LE)[[1]] == i, "Raw", "Women"] <- LifeTableChiangIIadj(popW1417[i,
        ], mortW[i, ])$EV[1]
}

# Smoothed LE is calculated by the expression 2.5
PoDsMen <- apply(ResulModelM$sims.list$P, c(2, 3), mean)
PoDsWomen <- apply(ResulModelW$sims.list$P, c(2, 3), mean)
dimnames(PoDsMen) <- dimnames(PoDsWomen) <- dimnames(popM)

for (i in row.names(popM)) {
    LE[dimnames(LE)[[1]] == i, "Smoothed", "Men"] <- LEfun(PoDs = PoDsMen[i,
        ], TT = 4)
    LE[dimnames(LE)[[1]] == i, "Smoothed", "Women"] <- LEfun(PoDs = PoDsWomen[i,
        ], TT = 4)
}
```

Results: plot figure

```r
# postscript('../Articulo_Mmodelv4/FigurasEV/Maps_EVc_HyM_0817_1x4_Greens2_PoDs.eps',
# width=9, height=4)
par(mfrow = c(1, 4), mar = c(1, 1, 1, 1), cex.axis = 0.6, cex.main = 0.9,
    cex.lab = 0.6)

for (Sex in c("Men", "Women")) {
    dat <- LE[, "Raw", Sex]
    dat[is.na(dat)] <- Inf
    datSinInf <- dat
    datSinInf[is.infinite(datSinInf)] <- NA

    dats <- LE[, "Smoothed", Sex]
    # dats[is.na(dats)] <- Inf

    septiles <- as.numeric(quantile(x = dats, probs = seq(0, 1, length.out = 8)[-c(1,
        8)], type = 4, na.rm = T))
    CortesFac <- factor(as.numeric(cut2(dat, cuts = c(-Inf, septiles, 1000,
        Inf))), levels = 1:8, labels = c(paste("[", format(min(dat), digits = 2,
        nsmall = 1), ", ", format(septiles[1], digits = 2, nsmall = 1),
        ")", sep = ""), paste("[", format(septiles[1], digits = 2, nsmall = 1),
        ", ", format(septiles[2], digits = 2, nsmall = 1), ")", sep = ""),
        paste("[", format(septiles[2], digits = 2, nsmall = 1), ", ", format(septiles[3],
            digits = 2, nsmall = 1), ")", sep = ""), paste("[", format(septiles[3],
            digits = 2, nsmall = 1), ", ", format(septiles[4], digits = 2,
            nsmall = 1), ")", sep = ""), paste("[", format(septiles[4],
            digits = 2, nsmall = 1), ", ", format(septiles[5], digits = 2,
```

```
            nsmall = 1), ")", sep = ""), paste("[", format(septiles[5],
            digits = 2, nsmall = 1), ", ", format(septiles[6], digits = 2,
            nsmall = 1), ")", sep = ""), paste("[", format(septiles[6],
            digits = 2, nsmall = 1), ", ", format(max(datSinInf, na.rm = T),
            digits = 2, nsmall = 1), ")", sep = ""), "Inf"))
    Cortes <- CortesFac
    valores <- as.numeric(CortesFac)
    # mypalette<-brewer.pal(length(levels(CortesFac)),'Blues') #'BrBG'
    mypalette <- paletaev5 <- RColorBrewer::brewer.pal(9, "Greens")[c(9,
        8, 7, 6, 5, 4, 3)]  #Paleta xa EV -> De oscuro a claro
    mipaleta <- c(mypalette, "white")
    fgs <- mipaleta[valores]

    plot(carto, col = fgs, xlab = "", ylab = "", axes = F)
    auxgrafcompesp <- matrix(c(levels(CortesFac), paste("(", as.numeric(table(factor(valores,
        levels = 1:8, labels = 1:8))), ")", sep = "")), nrow = 8, ncol = 2)
    legend("bottomright", legend = apply(auxgrafcompesp, 1, paste, collapse = " "),
        fill = mipaleta, border = c(mipaleta[1:7], "black"), cex = 0.8,
        bty = "n")
    if (Sex == "Men") {
        title("raw LE - Men", cex.main = 1)
    }
    if (Sex == "Women") {
        title("raw LE - Women", cex.main = 1)
    }
    rm(dat)
}

for (Sex in c("Men", "Women")) {
    dat <- LE[, "Smoothed", Sex]
    dat[is.na(dat)] <- Inf
    septiles <- as.numeric(quantile(x = dat, probs = seq(0, 1, length.out = 8)[-c(1,
        8)], type = 4, na.rm = T))
    CortesFac <- factor(as.numeric(cut2(dat, cuts = c(-Inf, septiles, Inf))),
        levels = 1:7, labels = c(paste("[", format(min(dat), digits = 2,
            nsmall = 1), ", ", format(septiles[1], digits = 2, nsmall = 1),
            ")", sep = ""), paste("[", format(septiles[1], digits = 2,
            nsmall = 1), ", ", format(septiles[2], digits = 2, nsmall = 1),
            ")", sep = ""), paste("[", format(septiles[2], digits = 2,
            nsmall = 1), ", ", format(septiles[3], digits = 2, nsmall = 1),
            ")", sep = ""), paste("[", format(septiles[3], digits = 2,
            nsmall = 1), ", ", format(septiles[4], digits = 2, nsmall = 1),
            ")", sep = ""), paste("[", format(septiles[4], digits = 2,
            nsmall = 1), ", ", format(septiles[5], digits = 2, nsmall = 1),
            ")", sep = ""), paste("[", format(septiles[5], digits = 2,
            nsmall = 1), ", ", format(septiles[6], digits = 2, nsmall = 1),
            ")", sep = ""), paste("[", format(septiles[6], digits = 2,
            nsmall = 1), ", ", format(max(dat), digits = 2, nsmall = 1),
            ")", sep = "")))
    Cortes <- CortesFac
    valores <- as.numeric(CortesFac)
    # mypalette<-brewer.pal(length(levels(CortesFac)),'Blues') #'BrBG'
    mypalette <- paletaev5 <- RColorBrewer::brewer.pal(9, "Greens")[c(9,
```

```
        8, 7, 6, 5, 4, 3)]   #Paleta xa EV -> De oscuro a claro
    mipaleta <- mypalette  #[length(mypalette):1]
    fgs <- mipaleta[valores]

    plot(carto, col = fgs, xlab = "", ylab = "", axes = F)
    auxgrafcompesp <- matrix(c(levels(CortesFac), paste("(", as.numeric(table(factor(valores,
        levels = 1:7, labels = 1:7))), ")", sep = "")), nrow = 7, ncol = 2)
    legend("bottomright", legend = apply(auxgrafcompesp, 1, paste, collapse = " "),
        fill = mipaleta, border = mipaleta, cex = 0.8, bty = "n")
    if (Sex == "Men") {
        title("smoothed LE - Men", cex.main = 1)
    }
    if (Sex == "Women") {
        title("smoothed LE - Women", cex.main = 1)
    }
    rm(dat)
}
```

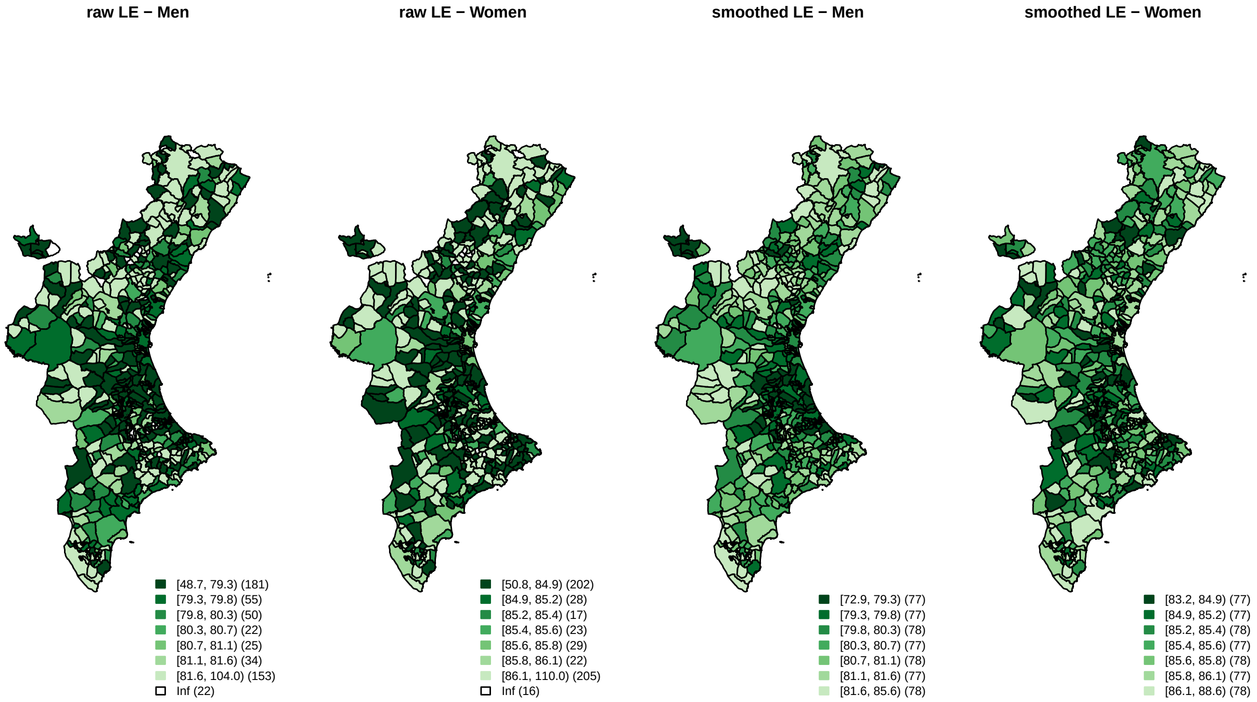


```
# dev.off()
```

Results: extreme values

```
round(diff(range(LE[, "Smoothed", "Men"])), 1)
```

```
## [1] 12.7
```

```
round(diff(range(LE[, "Smoothed", "Women"])), 1)
```

```
## [1] 5.5
```

```r
LE[LE == Inf] <- NA
round(diff(range(LE[, "Raw", "Men"], na.rm = T)), 1)
```

```
## [1] 55.3
```

```r
round(diff(range(LE[, "Raw", "Women"], na.rm = T)), 1)
```

```
## [1] 59.2
```
